# Exploring EZH2-Proteasome Dual-Targeting Drug Discovery through a Computational Strategy to Fight Multiple Myeloma

**DOI:** 10.3390/molecules26185574

**Published:** 2021-09-14

**Authors:** Filipe G. A. Estrada, Silvia Miccoli, Natália Aniceto, Alfonso T. García-Sosa, Rita C. Guedes

**Affiliations:** 1 Research Institute for Medicines (iMed.ULisboa), Faculty of Pharmacy, Universidade de Lisboa, 1649-003 Lisbon, Portugal; filipe.estrada@campus.ul.pt (F.G.A.E.); silvia.miccoli678@edu.unito.it (S.M.); 2 Department of Pharmaceutical Sciences and Medicines, Faculty of Pharmacy, Universidade de Lisboa, 1649-003 Lisbon, Portugal; 3 Institute of Chemistry, University of Tartu, Ravila 14a, 50411 Tartu, Estonia; 4 Department of Drug Science and Technology, University of Turin, Via Verdi 8, 10124 Torino, Italy

**Keywords:** multiple myeloma, polypharmacology, multitargeting (advantages), EZH2, Proteasome 20S, molecular docking, machine learning, QSAR, molecular dynamics

## Abstract

Multiple myeloma is an incurable plasma cell neoplastic disease representing about 10–15% of all haematological malignancies diagnosed in developed countries. Proteasome is a key player in multiple myeloma and proteasome inhibitors are the current first-line of treatment. However, these are associated with limited clinical efficacy due to acquired resistance. One of the solutions to overcome this problem is a polypharmacology approach, namely combination therapy and multitargeting drugs. Several polypharmacology avenues are currently being explored. The simultaneous inhibition of EZH2 and Proteasome 20S remains to be investigated, despite the encouraging evidence of therapeutic synergy between the two. Therefore, we sought to bridge this gap by proposing a holistic in silico strategy to find new dual-target inhibitors. First, we assessed the characteristics of both pockets and compared the chemical space of EZH2 and Proteasome 20S inhibitors, to establish the feasibility of dual targeting. This was followed by molecular docking calculations performed on EZH2 and Proteasome 20S inhibitors from ChEMBL 25, from which we derived a predictive model to propose new EZH2 inhibitors among Proteasome 20S compounds, and vice versa, which yielded two dual-inhibitor hits. Complementarily, we built a machine learning QSAR model for each target but realised their application to our data is very limited as each dataset occupies a different region of chemical space. We finally proceeded with molecular dynamics simulations of the two docking hits against the two targets. Overall, we concluded that one of the hit compounds is particularly promising as a dual-inhibitor candidate exhibiting extensive hydrogen bonding with both targets. Furthermore, this work serves as a framework for how to rationally approach a dual-targeting drug discovery project, from the selection of the targets to the prediction of new hit compounds.

## 1. Introduction

Multiple myeloma (MM) is a neoplastic plasma-cell disorder characterised by the clonal proliferation of plasma cells producing a monoclonal immunoglobulin, with devastating complications such as bone diseases, hypercalcemia, renal failure, anemia, and infections. It accounts for approximately 1% of neoplastic diseases and represents around 10–15% of all haematological malignancies diagnosed in developed countries [1]. Despite the remarkable advances in the MM treatment paradigm in the last decades, fostered by the approval of numerous therapies and therapeutic agents [2] and by deeper understanding of the pathogenesis of the disease, it is still considered incurable (and fatal) and the majority of MM patients exhibit primary treatment resistance or relapse during their lifetime. Multiple combinations of proteasome inhibitors (PIs), immunomodulatory drugs, monoclonal antibodies, histone deacetylase inhibitors, and more recently, immune checkpoint inhibitors, have led to the inception of multiple clinical options improving overall and progression-free survival [2], but the sobering reality is that most patients do not effectively benefit from these therapeutic strategies, which makes the development of new drugs and innovative therapeutic approaches urgent.

MM is a complex and multifactorial disease, and it is now clear that multifactorial diseases, such as cancer [3], neurodegenerative [4], infectious [5], and inflammatory diseases, might require a more complex therapeutic intervention that targets a given biological system as a whole [6].

Simultaneous inhibition of multiple targets is an established therapeutic strategy able to improve the efficacy, either additively or synergistically, while being less prone to the emergence of drug resistance mutations, and reducing adverse reactions, resulting in a superior clinical activity profile when compared to single-agent therapies, and may be a novel opportunity in the treatment of MM [7]. The challenge lies in discovering the appropriate targets and compounds with an appropriate multitarget profile, which are two of the main hurdles in the discovery and clinical development of these drugs.

An established target in the treatment of MM is the ubiquitin proteasome system (UPS), a highly complex, tightly controlled, and conserved pathway crucial for protein degradation and maintenance of cellular homeostasis [8]. The UPS is involved in the control of multiple cellular processes including cell cycle control, transcription, DNA damage repair, protein quality control, and antigen presentation and it is therefore not surprising that defects in this pathway have been associated with a number of pathologies, including haematological malignancies such as MM [9].

Proteasome 20S (P20S), UPS’ key particle, plays a critical role in the pathogenesis and proliferation of MM disease since plasma cells produce large amounts of immunoglobulin and are therefore particularly sensitive to the dysregulation of protein degradation. Particularly, PIs are one of the most important classes of chemotherapeutic drugs that have emerged for the treatment of MM and mantle cell lymphoma in the past two decades, and are currently the cornerstone drugs in the treatment of these haematological malignancies [10,11,12]. Three antitumour drugs in this class of PIs have been approved by the United States Food and Drug Administration (FDA) and European Medicines Agency (EMA): first-in-class bortezomib (Velcade^®^), carfilzomib (Kyprolis^®^), and ixazomib (Ninlaro^®^) [11,12]; second-generation PIs, as well as other classes, are still under clinical development (https://clinicaltrials.gov/, visited on 22 July 2021). Chemoresistance to PIs is pointed out as the key driver for the failure of clinical management, but the genetics and epigenetics that play a role in such chemoresistance are still unclear.

A growing body of evidence suggests that epigenetics-targeting drugs could be an important strategy to successfully treat MM. In 2011, MM sequencing and transcriptomics [13] revealed that mutations and copy number variations, as well as dysregulation in the expression of epigenetic modifiers, are characteristic features of MM. In the past decade, several studies have suggested epigenetic mechanisms that involve the control of gene expression by factors other than the individual DNA sequence, via DNA methylation, histone modifications, and noncoding RNAs as important contributing factors to drive malignant MM phenotypes [14]. Therefore, epigenetic mechanisms were proposed as contributing to the clonal heterogeneity and plasticity observed in MM. Recent studies support epigenetic plasticity in MM and suggest it to be an underlying mechanism regulating myeloma cell phenotypic diversity and drug resistance [15,16].

The epigenetic regulator EZH2 histone methyltransferase has been shown to be misregulated due to overexpression and mutations in tumours with different origins, such as ovarian cancer [17], prostate cancer [18], glioblastoma [19], medulloblastoma [20], germinal center derived B-cell lymphomas [21], and also in MM, causing trimethylation of H3K27, H3K27me3 [15]. These studies suggest that the aberrant activity of EZH2 is a key player in tumour formation, progression, metastasis, and therapeutic response. Pawlyn et al. demonstrated the independent deleterious effect of EZH2 overexpression on MM treatment clinical outcomes, making it an attractive target [15].

**Figure 1:**
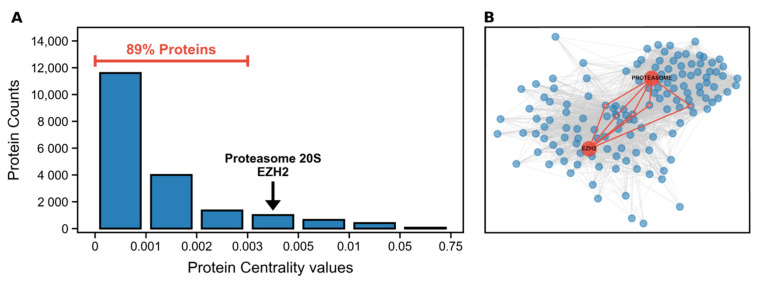
(**A**) Distribution of centralities across the whole human proteome, highlighting the bin containing P20S and EZH2; and (**B**) protein signaling network of EZH2, P20S, and all the signaling paths that connect them, with a maximum length of 4 (i.e., 2 intermediate nodes between the two target proteins). This plot contains a total of 1063 paths and 139 proteins. The blue points represent neighbours of EZH2 and P20S, grey lines indicate signaling relationships between proteins, and red lines highlight connections to immediate neighbours shared by the two key proteins.

Interestingly, inhibiting EZH2 has been shown to sensitise P20S to the action of inhibitors [22], which prompted us to explore a new strategy to target both proteins as a way to induce cell death in cancer tissues. Both P20S’ β5 subunit and EZH2 are among the top 10% most central proteins in the human proteome (Figure 1A). High degree of centrality has been associated with essential proteins in a network [23]. However, this measures *local* importance, in a specific biological context. In addition to this, EZH2 and P20S are also very close to each other within the human signaling network annotated in OmniPath (https://omnipathdb.org/) (Figure 1B). As a drawback, due to their high degree of centrality, the targeting of either of these two proteins is also prone to off-target effects in normal tissue; however, these are relatively less likely seeing as EZH2 and P20S have comparatively high local-to-global importance ratio (i.e., are placed at 50% of the ranking of degree-to-betweenness ratio). Nonetheless, dual-targeting therapy is in itself an important strategy to offset possible side effects as they allow lowering the therapeutic dose [24,25,26] and have been associated with a better toxicity–efficacy balance [27].

P20S has already been the focus of different dual-targeting strategies for drug development whereby compounds were developed to directly modulate P20S and, simultaneously, HDAC6 [28], HDAC1 [29], and fatty acid synthase [30], among others. However, to our knowledge, there are no known studies focusing on the dual targeting of P20S and EZH2. In face of the reported evidence that shows modulating one target that has a transferable effect into the other target, it is important to explore the feasibility of developing dual inhibitors for these two targets. Currently there is a considerable amount of (single agent) active compounds reported against P20S and EZH2 which provide a good starting point for such an endeavour.

Herein, we carried out an exploratory computational effort combining molecular docking, interaction profile analysis, machine learning, and molecular dynamic approaches that aims to be a framework for identifying multitarget inhibitors that can overcome resistance and hopefully improve antineoplastic activity.

## 2. Results

### 2.1. An Overview of the Chemical Space of Known EZH2 and P20S Inhibitors

**Figure 2:**
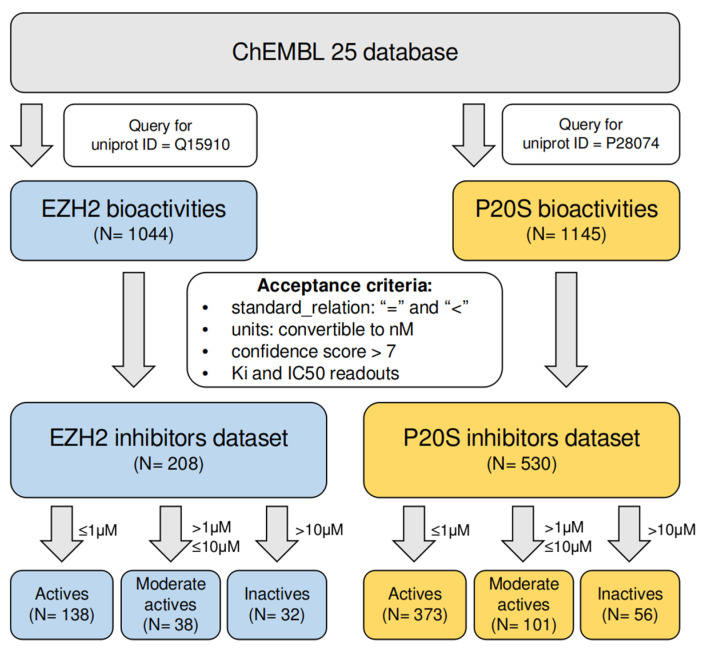
Overview of the assembly process for the EZH2 inhibitors and the P20S inhibitors datasets.

The first step in designing new molecules against any target is to consider what is currently known on the relationship between the structure and activity for said target. Additionally, in a dual-targeting problem, it is important to scope any overlapping structural patterns between inhibitors of the two targets. This allows promoting that any proposed dual-inhibitor candidates are both novel and viable. To this end we extracted all bioactivity data from ChEMBL 25, annotated for P20S (here we restricted the retrieval to data referring to the β5 subunit) and EZH2 proteins. These two datasets were subsequently cleaned and filtered using criteria listed in Figure 2 (see Methods section for more details) and the final datasets were composed of 208 and 530 compounds in the EZH2 inhibitors dataset and the P20S inhibitors dataset, respectively. These were divided into three classes each (actives, moderate actives, and inactives). The complete dataset assembly is summarised in the workflow shown below (Figure 2).

**Figure 3:**
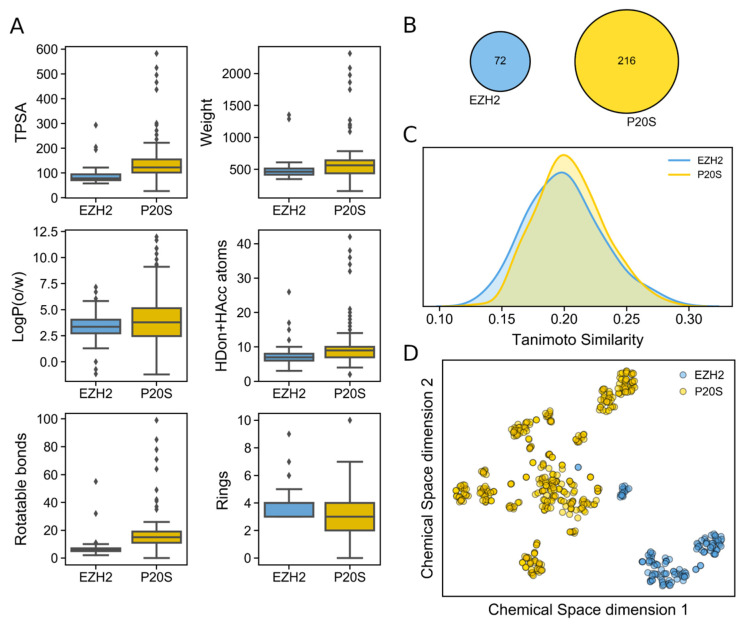
Physicochemical profile and chemical space of EZH2 and P20S inhibitors; (**A**) distribution of six key physicochemical properties calculated in MOE 2019.01; (**B**) overlap in Murcko scaffolds; (**C**) maximum Tanimoto similarity between each EZH2 inhibitor and all P20S inhibitors (yellow) and maximum Tanimoto similarity between each P20S inhibitor and all EZH2 inhibitors (blue); and (**D**) chemical space representation using t-SNE over Morgan Fingerprints.

While we had previously carried out a comprehensive analysis on the known chemical space of P20S inhibitors [8], to our knowledge, a similar study does not exist for EZH2 inhibitors. We therefore conducted a summary analysis of several properties and the chemical space of EZH2 inhibitors and compared them with those of P20S (summarised in Figure 3).

**Figure 4:**
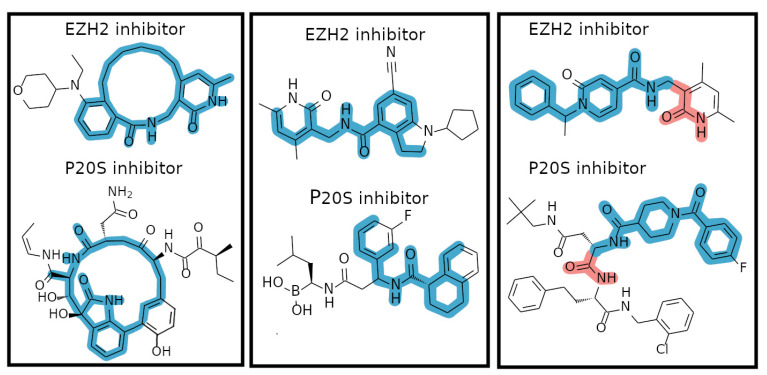
Representative examples of identified shared substructures between EZH2 and P20S inhibitors. Strictly mapped bonds/atoms are shown in blue (where aromatic only map to aromatic and aliphatic to aliphatic), and relaxed mappings (aromatic atoms can be matched to equivalent aliphatic atoms) are shown in red.

Comparing the physicochemical profile of both sets of inhibitors shows that EZH2 inhibitors tend to have smaller polar surface (TPSA) values as well as fewer rotatable bonds. Other than this, both types of inhibitors show similar distribution of lipophilicity, total H-bonding atom count, molecular weight, and ring count (Figure 3A). However, P20S inhibitors typically show more extreme values in all six properties analysed. Upon inspecting the Murcko scaffold content in both datasets, we identified no overlap between P20S and EZH2 inhibitors (Figure 3B). A total of 216 and 72 Murcko scaffolds were identified in each dataset, respectively, which show identical diversity (0.53 and 0.58 scaffold-per-compound ratio). Considering Murcko scaffolds are biased towards the presence of rings, we analysed the maximum common substructures between them. This revealed 46 unique pairs of EZH2-P20S inhibitors that share large substructures (i.e., >15 atoms) between EZH2 and P20S compounds, where aromatic atoms are only mapped to aromatic atoms. The three most common shared scaffolds are shown in Figure 4, which indicate that a remarkably large substucture of the depicted EZH2’s inhibitors is shared by some P20S inhibitors. This was encouraging for the purpose of our dual-targeting approach.

The analysis of the similarity between both datasets revealed that the most similar compounds between EZH2 and P20S inhibitors show around 30% similarity, and typically the highest-similarity pairs between these two datasets have around 20% similarity (Figure 3C). This reveals that both sets of inhibitors are considerably different to each other. In line with previous analyses, the distribution in chemical space for each set of inhibitors is markedly distinct (Figure 3D); however, a few EZH2 compounds appear to be close to P20S’ chemical space.

### 2.2. Comparison of Binding Pockets in EZH2 and P20S

The analysis of the three-dimensional (3D) structures of proteins, and especially the inspection of binding sites, is paramount in drug discovery. It is widely recognised that seemingly unrelated related proteins may have similar binding sites able to bind chemically similar ligands. Thus, the similarity between binding sites of two different proteins can be a useful predictor of new pairs of targets amenable to multitargeting, or even for drug repositioning. Here we compared the pockets of EZH2 and P20S proteins to ascertain similarities that may be significant in the interaction of the ligands with both targets, such as accessible surface area, hydrophobicity and charge of the pockets, and shape, among others. Here we used the CT-L binding site for P20S (found at the interface between the β5 and β6 subunits) which is one of three different binding sites of P20S. For EZH2 we used the catalytic pocket formed by the SET and SAL domains.

**Figure 5:**
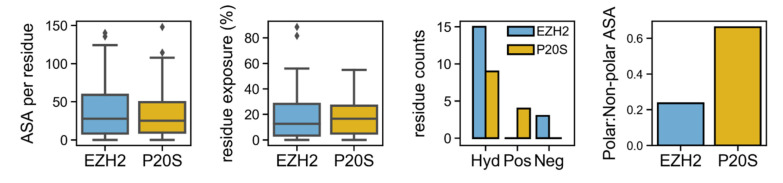
Assessment of accessible surface of binding pockets in EZH2 and P20S (β5 pocket). ASA is defined as the accessible surface area (Å^2^) and expresses the area around a residue that is exposed to the solvent. Residue exposure measures how much (in %) of a residue’s surface is exposed. Additionally, the number of residues according to type (hydrophobic (Hyd), positive (Pos), and negative (Neg)) is shown and, complementarily to this, the ratio of polar:nonpolar residues is also shown.

The binding pocket in P20S has a total surface of 1411.9 Å^2^, which is marginally smaller than the 1421.6 Å^2^ seen for EZH2’s pocket. The same trend is seen for the distribution of accessible surface area (ASA) values observed across pocket residues for either protein. However, while P20S’ pocket only has one highly exposed residue (>50% exposure), EZH2’s pocket shows four highly exposed residues. In addition, EZH2’s pocket has a much more hydrophobic surface as seen by the residue counts for hydrophobic versus charged atoms, as well as by the relative amount of polar surface in the total pocket surface (0.2 for EZH2 vs. 0.6 for P20S) (Figure 5). Furthermore, EZH2’s pocket has 60 hydrophobic contact points and P20S has only 41, as determined by MOE’s SiteFinder (identified pocket residues are listed in Supplementary Materials Table S1).

**Figure 6:**
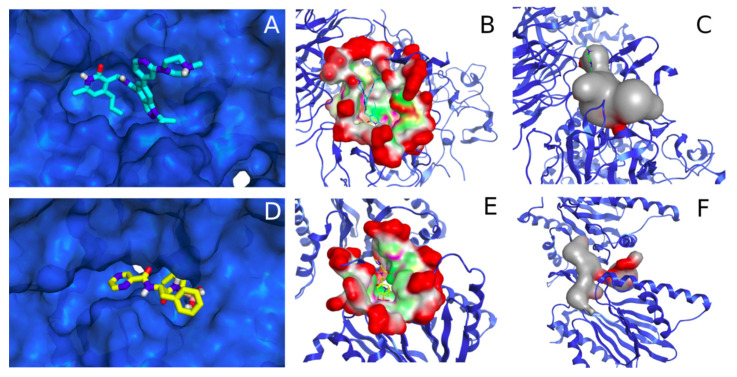
Binding pockets in EZH2 and P20S, respectively. The top row refers to EZH2’s pocket while the bottom row refers to P20S; (**A**,**D**) X-ray ligand in the binding pocket (GSK343 in cyan and bortezomib in yellow); (**B**,**E**) ligand in the binding pocket whose surface is colour-coded according to exposed (red), hydrophobic (green), and polar (pink) regions; (**C**,**F**) volume contained within the boundaries of the pocket cavity, hydrophilic (red), hydrophobic (grey). The detected pockets are defined in Supporting Table S1.

The binding pocket of EZH2 is more voluminous (201 versus 161 alpha-spheres, calculated in MOE’s SiteFinder) and more spherical in shape (Figure 6C), whereas P20S’s pocket appears to be narrow, elongated, and formed of two contiguous subpockets (Figure 6F). Despite these differences, polar and nonpolar regions are spread throughout the two pockets which could potentially accommodate one single molecule in establishing multiple polar contacts in both pockets (Figure 6B,E).

**Figure 7:**
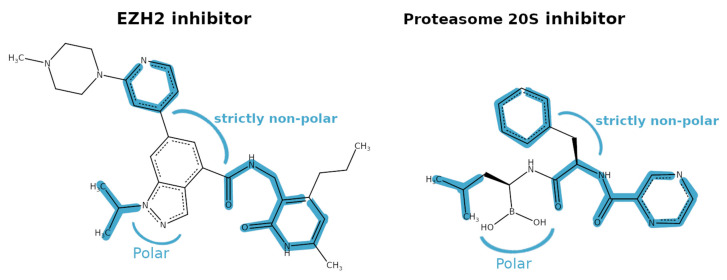
Hypothesis for 2D similarities between EZH2 and P20S X-ray ligands (GSK343 and bortezomib, respectively), shown from left to right, where the blue regions highlight equivalent or very similar regions of both molecules.

When inspecting the two X-ray inhibitors (GSK343 in EZH2 and bortezomib in P20S) we observed some common substructures and possibly overlapping polar and nonpolar regions that hint at the possible binding of both compounds into both pockets (Figure 7).

**Figure 8:**
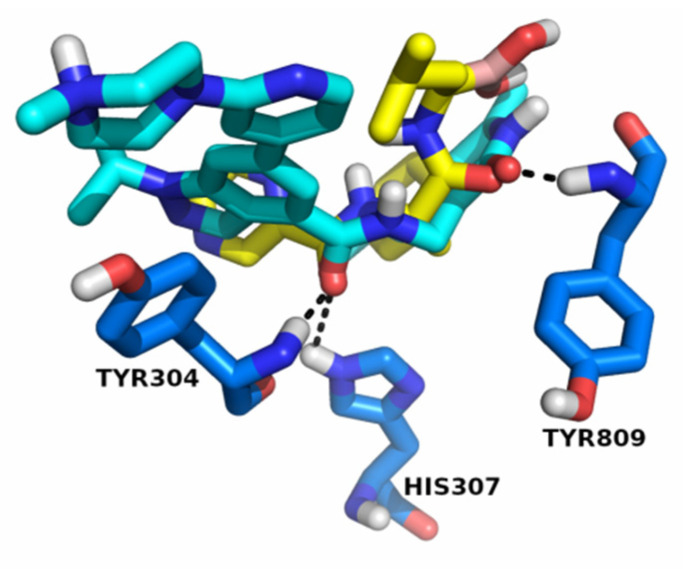
GSK343’s X-ray pose (cyan) and bortezomib (yellow) in EZH2’s binding pocket. The three key H-bonding residues (TYR304, HIS307, and TYR809) in this pocket are shown in blue, and H-bonds are identified as dashed black lines.

In order to gauge the viability of EZH2 and P20S inhibitors to act upon the two targets we ran a preliminary docking calculation of the P20S’s ligand (bortezomib) in EZH2’s pocket and the resulting pose obtained for bortezomib is overlaid with EZH2’s X-ray ligand, as shown in Figure 8. The pose revealed that bortezomib is particularly well superposed against the portion of the EZH2’s inhibitor (GSK343) that is embedded into the pocket (which is arguably more meaningful than an overlay with the “free” end of the X-ray ligand which is exposed to the solvent). Notably, one of the two amides in bortezomib is closely aligned with the amide in GSK343, with both carbonyls placed almost precisely at the same position (0.3 Å distance). This means that one bortezomib’s carbonyl is able to establish dual H-bonding with HIS307 and TYR304 which is also seen for GSK343. Similarly, the carbonyl in bortezomib’s other amide is at 0.6 Å from the carbonyl of GSK343. Additionally, the bortezomib’s pyrazine ring is in close proximity to the indazole ring (pyrazole moiety) in GSK343, with two nitrogens (one from each compound) placed at 1.59 Å from each other. Considering that TYR304 has been described to stabilise ligands in the binding pocket, and TYR809 is an important residue to which the S-adenosyl methionine cofactor binds [31], this indicates that it would be realistic to envision one single molecule that binds both pockets.

**Figure 9:**
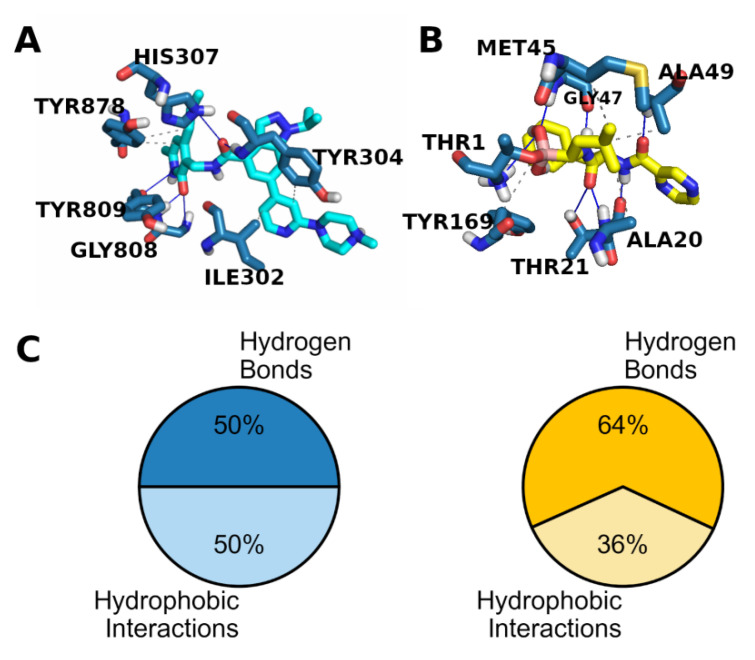
Interaction profile in the pockets of EZH2 (5WFC) and P20S (5LF3) X-ray structures (hydrogen bonds are marked by a blue line and hydrophobic interactions by a grey dashed line); (**A**) protein–ligand interactions between EZH2 and its X-ray ligand GSK343 (cyan); (**B**) protein–ligand interactions between P20S and its X-ray ligand bortezomib (yellow); and (**C**) distribution of interaction types found in EZH2 (5WFC) and P20S (5LF3) X-ray structures, shown in blue and yellow pie charts, respectively.

The same analysis for the P20S β5 pockets is not as clear cut because bortezomib is covalently bound, and we did not carry out any covalent docking. Nevertheless, binding at the β5 (CT-L) site appears to require a multitude of hydrogen bonds owed to the several H-bonding groups placed in close sequence across the length of the pocket [32] as is evident in Figure 9B. GSK343 clearly has numerous hydrogen bond acceptors and donors throughout its structure and, specifically, it has an amide that could conceivably be placed in the same location of either of the two amides in bortezomib (Figure 9A). Overall, hydrogen bonding appears to play a central role in binding to both EZH2 and P20S, evident by how the corresponding X-ray ligands bind (Figure 9A,B) as well as by the amount of hydrogen bonds observed among all bonds (Figure 9C).

### 2.3. Molecular Docking of EZH2 and P20S Inhibitor Datasets to Select Dual-Binding Compounds

#### 2.3.1. Validation of the Molecular Docking Calculations

Ligand docking has been successfully used for drug discovery in the last decades to predict the three-dimensional structure and “binding free energy” of the complex formed by a receptor, usually a protein, and a small ligand [33]. When applied iteratively to a library of small molecules (structure-based virtual screening), each member of the library is docked into the receptor, assigned a predicted binding energy, and ranked accordingly. Despite its widespread and successful use, docking suffers from a strong disadvantage: the ability to tackle the intrinsic flexibility of proteins is absent or limited. This disadvantage can limit the predictive potential of this technique and for this reason it is paramount to validate the docking accuracy for each individual system that we want to study. Correlation between experimental activity and score, poses inside the protein binding pocket, and protein–ligand interaction profiles must be critically evaluated. 

Before any proper docking calculations, the validation of the docking protocol was performed through self-docking of the X-ray ligands of EZH2 (5WFC) and P20S (5LF3) in their own original binding pocket and the obtained pose was compared with that of the X-ray structures obtained experimentally. All the P20S and EZH2 ligands for which there was an X-ray structure in the respective proteins were also docked in order to perform a simplified “cross-docking” that would allow us to reinforce our validation and confidently proceed with our work. For EZH2 RMSD values between 1.13 Å and 7.63 Å were obtained, of which three of the seven tested ligands showed RMSD < 2 Å. For P20S we obtained RMSD values between 1.52 Å and 4.51 Å (higher values in this protein are acceptable given we are comparing noncovalent poses with covalent X-ray positions).

Self-docking and cross-docking simulations were performed on the active sites of the two proteins mentioned above using Schrödinger’s Glide with extra precision (XP) module (Schrödinger LLC, v.2019) (more details can be found in the Methods Section).

#### 2.3.2. Construction of a Predictive Model for EZH2 and P20S Inhibitors

After the docking protocol was validated we employed it to dock the two ChEMBL datasets against P20S’ β5 and EZH2’s SET-SAL binding sites, in order to identify compounds that showed a good affinity for both P20S and EZH2. Docking simulations produced two important outputs: docking scores and Protein-Ligand Interaction Fingerprints (PLIFs) which were subsequently used to build a predictive model to select dual inhibitors.

After concluding the molecular docking calculations, we used these results to build a predictive model/function, testing different approaches, that can accurately separate actives from inactives, and their “actives screening” performance was assessed through enrichment curves (Supporting Figure S1). Essentially, for any given screening function used, steeper acceptance criteria applied to select actives should produce higher percentages of actives among selected compounds. The collection of the various measurements of selection criteria and their corresponding retrieval of actives corresponds to an enrichment curve.

First, we assessed the screening performance produced by using the docking score and ligand efficiency (LE = docking score/# heavy atoms), when applied to the ChEMBL datasets, and concluded that these two parameters do not correlate reliably with activity, through the analysis of the corresponding enrichment curves. In other words, as the threshold value for compound selection becomes more stringent the % of captured actives actually decreases (Supporting Figure S1). As an alternative, we tested the use of similarity of PLIFs profiles between the X-ray ligand of the corresponding protein for which docking was run, and the docking poses of each ligand in the ChEMBL dataset, and we were able to get no enrichment for EZH2 and good enrichment for P20S (Supporting Figure S1).

**Figure 10:**
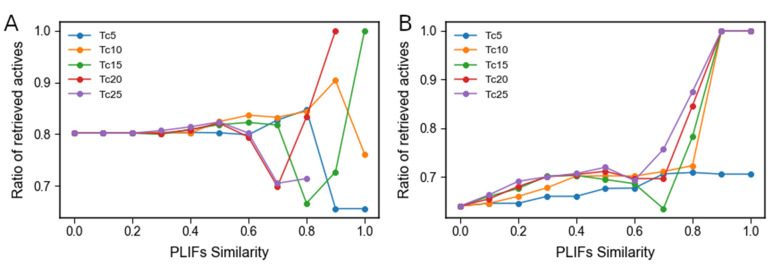
Plots of the enrichment curves calculated using the similarity of residue contacts between the ligands docked poses and the X-ray ligand. The curves are labeled in the plots as “TcN” where “Tc” refers to the Tanimoto coefficient which was the similarity function applied to the PLIFs profiles, and “N” is replaced by the amount of top N most frequent contacts observed in the ChEMBL datasets screened; (**A**) enrichment curves for the EZH2 inhibitors dataset, among which the yellow curve (using 10 residues) was selected to filter new predicted EZH2 inhibitors among known P20S inhibitors; and (**B**) enrichment curves for the P20S inhibitors dataset, among which the red curve (using 20 residues) was selected to filter new predicted P20S inhibitors among known EZH2 inhibitors.

**Figure 11:**
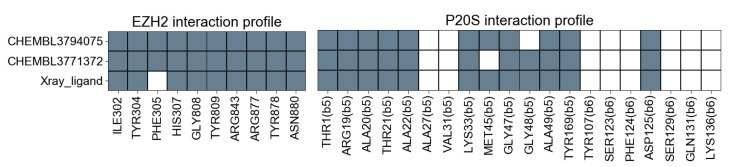
PLIFs profile of the contacts between the X-ray ligand and the compounds selected, and the proteins in the molecular docking simulations, calculated in Schrödinger. The profiles for the two docking hits correspond to the top-scoring pose obtained with Glide.

Given PLIFs similarity was initially calculated from all available residues in the full contacts matrix, we next optimised the calculation of PLIFs similarity by building enrichment curves with the 5, 10, 15, 20, and 25 most frequently engaged residues across all ligands (Figure 10). For the EZH2 the use of the 10 most frequently engaged residues across all compounds to calculate PLIFs similarity produced the best enrichment as it is the only one with a steady increase until 0.9 similarity reaching a maximum enrichment value of 90%. The final measured point in this curve shows a drop in performance which means that one should exclude compounds that show 100% PLIFs similarity to the X-ray ligand. This decrease in performance can likely be a result of very sparse coverage, which might lead to covering a few compounds and some of them just happening to be inactive (despite being well placed in the binding pocket) (Figure 10A). For P20S the best curve was built using 20 residues (producing a maximum enrichment of 100%), given the fact that five residues do not produce a high enough enrichment for actives (maximum at 70%) and 10 residues show a good enrichment but a low coverage at high enrichment values (only 34 compounds covered at >0.8 similarity). Using 15 residues produces an undesirable steady drop in performance between 0.4 and 0.7 similarity. The curve obtained from 25 residues produces good enrichment, comparable to the 20, but shows a slightly more pronounced drop in performance at 0.5 similarity than that of the 20-residue curve (Figure 10B). The residues used for both selected similarity functions are listed in Figure 11.

After the curves were selected, the following condition was defined to predict dual-inhibitor compounds:

0.8 < PLIFsim (EZH2) ≤ 0.9 and PLIFsim (P20S) ≥ 0.9

**Figure 12:**
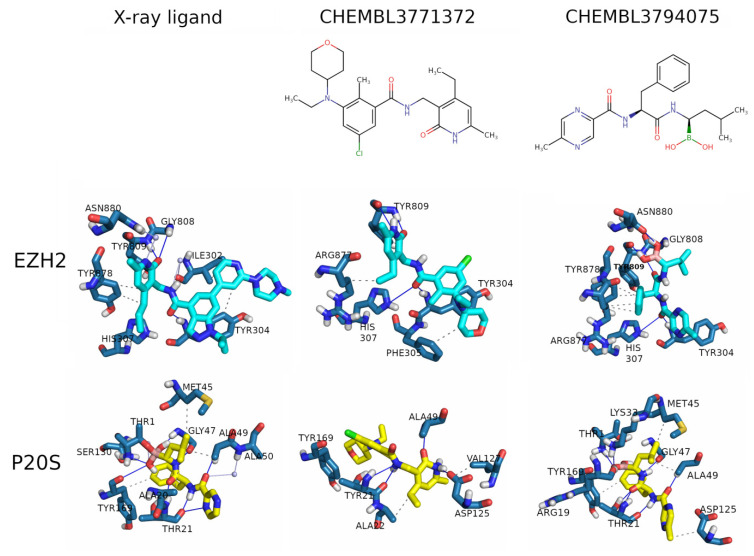
Representation of the 2D structure of the docking hits (**top row**), and 3D placement of the ligands (X-ray and docking hits) in the pockets of EZH2 (**middle row**) and P20S (**bottom row**). Residues are shown in blue, ligands are shown in yellow and cyan, hydrogen bonds are represented in dark blue, and the dotted grey lines represent the hydrophobic interactions. The white spheres are water molecules and the lines connecting to them represent water bridges. Interactions calculated with PLIP tools, which entails a few differences to the interaction profiles produced by Schrodinger in Figure 11, related to how hydrophobic interaction are detected.

From the EZH2 inhibitors dataset and P20S inhibitors dataset, two ligands fulfilled this criterion and were selected, one from each dataset (CHEMBL3794075 and CHEMBL3771372, shown in Figure 12).

#### 2.3.3. Interactions of the Selected Docking Hits

To assess the potential of the two selected compounds from the molecular docking simulations we identified for each of these compounds the specific interactions that they established with each of the proteins. We compared these data with information obtained from X-ray cocrystallised structures with known inhibitors as well as with results already gathered from the several molecular docking simulations performed by other groups to gain insight into EZH2 [34,35,36,37,38,39,40] and P20S inhibition [32,41,42,43,44,45,46].

The interaction profiles (i.e., PLIFs) of the two docking hits with both targets alongside the interactions established by the corresponding X-ray ligands is provided in Figure 11, including only the key residues that are a part of the two similarity functions selected previously. The comparison of the PLIFs similarity calculated between each docking hit and the corresponding X-ray is shown in Table 1, showing that both ligands show highly similar PLIFs profiles to the corresponding X-ray ligand, in both EZH2 and P20S.

The docked pose of each ligand in both proteins was analysed using PLIP tools [47] to extract all established interactions with each target, and the obtained interaction profiles are shown in Figure 12. 

Regarding the docking in EZH2, as expected CHEMBL3771372 (a known EZH2 inhibitor) shows a high resemblance with the pose of EZH2’s X-ray ligand, where the shared substructure between the two is closely overlaid (Supporting Figure S2). Despite being quite different structurally from the previous two, CHEMBL3794075 is placed deep inside the binding pocket (Supporting Figure S2). The X-ray cocrystallised ligand forms hydrophobic interactions with residues ILE302, TYR304, and TYR878. However, CHEMBL3771372 only shows hydrophobic interactions with PHE305 and ARG877, and CHEMBL3794075 with ARG877 and TYR878. This may be caused by the smaller size of both hit compounds. The X-ray cocrystallised ligand establishes H-bonds with TYR304, GLY808, and TYR809, of which two are shared with CHEMBL3771372 (TYR304 and TYR809). On the other hand, CHEMBL3771372 forms an additional H-bond with HIS307. CHEMBL3794075 shows all H-bonds observed for the X-ray cocrystallised ligand (TYR304, GLY808, and TYR809) with additional H-bonding to HIS307 and ASN880. Additionally, the X-ray cocrystallised ligand shows two concerted H-bonds between the same carbonyl oxygen and both TYR304 and HIS307, which is also observed in both docking hits. Zhou et al. [29] unveiled a series of pyrrole 3-carboxamide in which hydrophobic interactions with TYR111 (TYR304 in our case), TYR558 (TYR851 in our case), and PHE562 (PHE855 in our case), as well as double hydrogen bonds with TRP521 (TYR809 in our case), are critical for EZH2 inhibitory activity. In the residue correspondence, even though the residues are not of the same type, the OH donor of TYR809’s side chain is very close to the NH donor of TRP521’s side chain, allowing for the previously identified double bonding. Likewise, the EZH2 inhibitors in clinical trials all have similar and unique pyridone moieties, which are required to enhance binding to the EZH2 SET domain in a cofactor-competitive manner via forming two hydrogen bonds with TRP521/TYR809.

Given the profile of interactions established, even though CHEMBL3771372 is closely overlaid with the X-ray cocrystallised ligand, CHEMBL3794975 shows more hydrogen bonds that span the full length of the molecule, which makes it a more promising candidate for the development of a dual EZH2-P20S inhibitor. The higher number of hydrogen bonds may also provide specificity to this binding pocket.

Regarding the docking in P20S, and similarly to what was observed for EZH2, the known P20S inhibitor CHEMBL3794075 shows almost perfect overlap with the pose of P20S’ X-ray cocrystallised ligand (Supporting Figure S3). CHEMBL3771372 does not occupy the pocket as deeply but overlaps a large part of the X-ray cocrystallised ligand (with the hydrogen of the central amide at 0.46 Å of the equivalent hydrogen in the X-ray cocrystallised ligand).

The X-ray cocrystallised ligand forms hydrophobic interactions with residues ALA20, MET45, ALA49, and TYR169, with no overlap with CHEMBL3771372 (ALA22 and VAL127), and with two contacts in common with CHEMBL3794075 (MET45, ALA49, ASP125, and TYR169). Furthermore, the X-ray cocrystallised ligand establishes H-bonds with THR1, THR21, GLY47, ALA49, and TYR169, two of which are also observed for CHEMBL3771372 (THR21 and ALA49) which additionally shows an H-bond with ASP125. CHEMBL3794075, however, shares three H-bonds with the X-ray cocrystallised ligand (THR1, THR21, and ALA49) and further interacts with ARG19, LYS33, and GLY47. Importantly, the X-ray cocrystallised ligand forms a covalent bond between THR1 and the boron atom, and CHEMBL3794075, which also has a boron atom, is positioned close to THR1. The key residues highlighted across different noncovalent molecular docking works and the X-ray observations, indicating THR1, THR21, GLY47, ALA49, TYR107, and ASP125 as key residues [41,43], are in line with the findings in this work.

Given the profile of interactions observed for P20S, CHEMBL3794975 shows more hydrogen bonds and almost perfectly emulates bortezomib’s X-ray crystal pose, which is a very encouraging outcome. Therefore, results in P20S also point to CHEMBL3794975 as a more promising candidate for the development of a dual EZH2-P20S inhibitor, making it the most promising dual-targeting candidate overall, when results from both targets are considered.

### 2.4. Construction of QSAR Models Using Machine Learning to Predict Dual-Targeting Inhibitors against P20S and EZH2

As a complement to the molecular docking-derived model (i.e., PLIFs similarity filter), we sought to train a machine learning model on the ChEMBL datasets used in the molecular docking section. To do so, we chose to use a decision tree algorithm for the excellent interpretability it allows and, to avoid a harsh single binary threshold between actives and inactives, we opted training with three classes (adding a “moderate activity” class to “actives” and “inactives”). We optimised modelling conditions for both targets by comparing the use of different feature sets, namely Morgan fingerprints, physicochemical descriptors (from MOE), PLIFs, and all three feature sets combined, and concluded that physicochemical descriptors led to the best performance in the test set. These models were all built with misprediction penalty weights inversely proportional to the class size. After physicochemical descriptors were selected as the optimal feature set, five schemes of misprediction penalty weights were tested (see Methods for details). The final model was selected based on the best test set performance, which was built using equal weights for both P20S and EZH2.

Overall, the two datasets produced models with high performance in correctly proposing actives, demonstrated by the high precision values seen in both models (0.84 for both EZH2 and P20S, respectively) as shown in Table 2. However, the decision tree algorithm struggled to separate moderate actives from inactives in the EZH2 model (as seen by the lower precision values for these two classes). In P20S’s model, inactives were successfully predicted (precision = 0.83) but the decision tree algorithm struggled to distinguish between moderate actives and the two other classes (precision = 0.44). This outcome recapitulates the limitations of binning data into fairly generic bins of activity, because indeed some moderate actives are practically active or practically inactive and so, being predicted as such might actually be correct when considering how we perceive activity.

Nevertheless, at this stage of compound screening one would be mostly interested in accurately proposing new actives (i.e., high precision for actives) given the fact that academic teams are often only able to purchase or synthesise a relatively small amount of hits. Therefore, our models were deemed well suited for such an application. 

To our knowledge there is only one work from 2011 on the use of machine learning (support vector machines, precisely) to predict inhibitors of proteasome 20S, but in this case the authors focus on *P. falciparum*’s protein [48]. There is also only one work, currently unpublished (in ChemRxiv) [49], where the authors have built a QSAR machine learning model (using support vector machines) of many epigenetic targets, one of which is EZH2. Their model was built on 304 compounds and, even though they do not mention how much of the data were used for training, the trained models they provide show that all 304 instances were used in model fitting. Therefore, their reported performance of 0.831 from 10-fold cross-validation cannot be directly compared to ours, which is measured on an external set of compounds. Differently from us, they report that Morgan Fingerprints yield the best predictive performance for EZH2 (but physicochemical descriptors have as high a performance).

Regarding the decision tree model built to predict EZH2 inhibitors (Supporting Figure S4), the water solubility (h_logS), molecular weight, and lipophilicity (SlogP) enabled a dramatic separation between actives and the rest of the compounds, leading to an enrichment of actives from 65.7 to 83.3%. These indicate that an EZH2 inhibitor is typically characterised by not being overly small (>350 g/mol) and by being highly lipophilic. This is in line with the fact that the surface of EZH2’s pocket is much more lipophilic than that of P20S (Figure 5).

**Figure 13:**
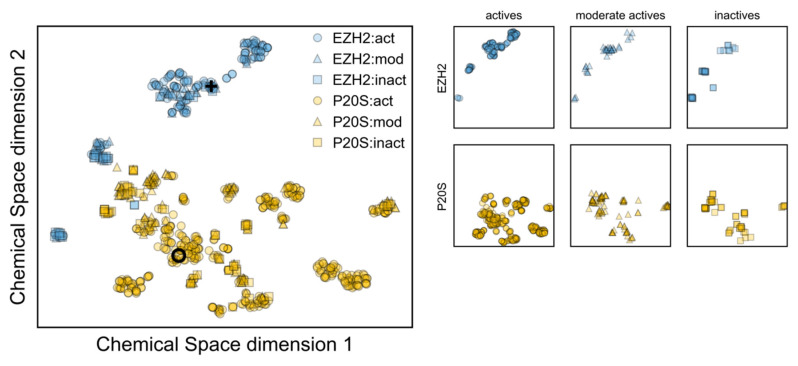
Chemical space of the EZH2 inhibitors dataset (blue) and the P20S inhibitors dataset (yellow). The black circle represents CHEMBL3794075 and the black cross marks CHEMBL3771372, the two hits originating from docking.

The decision tree for P20S inhibitors (Supporting Figure S5) revealed that total positive van der Waals surface area (PEOE_VSA_PPOS), the sum of H-donor strengths, and polar/hydrogen-bonding volume (i.e., vsurf_Wp5, the volume enclosed by the solvent-accessible polar surface [50,51]) contributed to a dramatic increase in the separation between actives and the rest of the compounds, leading to an enrichment of actives from 70.5 to 93.3%. This is in line with findings from X-ray data which indicate that binding to Proteasome 20S’ β5 pocket is heavily driven by H-bonding (Figure 9B,C and Figure 13) and is also in accordance with the fact that P20S’ pocket surface is much more polar than that of EZH2 (Figure 5 and Figure 6).

After the models were built, we next applied them to predict the EZH2 inhibition for P20S inhibitors and vice versa. From this analysis we obtained 12 predicted P20S inhibitors and 260 predicted EZH2 inhibitors (adding up to a total of 272 predicted dual inhibitors). Unfortunately, none of these QSAR hits coincide with the two hits proposed by molecular docking. CHEMBL3771372 (which is the known EZH2 inhibitor) was predicted as moderately active against P20S, and CHEMBL3794075 (the known P20S inhibitor) was predicted as inactive against EZH2. Faced with the lack of agreement between the machine learning predictions and the docking predictions, we opted to pursue further studies with the latter. This decision was justified by the very low overlap in chemical space between the two datasets (Figure 13), which indicates that any EZH2 compounds are likely outside the applicability domain of P20S’ model and *viceversa*. For this reason, the lack of agreement between the two prediction models was expected. Moreover, the limited applicability of each machine learning model to the other target’s chemical space means there is low confidence in any predictions made in this setting, which led us to proceed exclusively with the hits from docking.

### 2.5. Molecular Dynamics Studies to Investigate Dual-Inhibitor Hits Produced from Molecular Docking

After the docking hits were selected, the structures were prepared; in the case of the proteasome the two stacked β rings (consisting of seven β subunits each) were kept, and the simulation box for the systems was built, solvated, minimised, equilibrated, and then simulated during 200 ns.

The first analysis of the simulations was the total energy of the systems, to evaluate if the system is well minimised and equilibrated. The total energy during the simulation should be constant, which we have observed in all four molecular dynamics runs (Supporting Figure S6).

**Figure 14:**
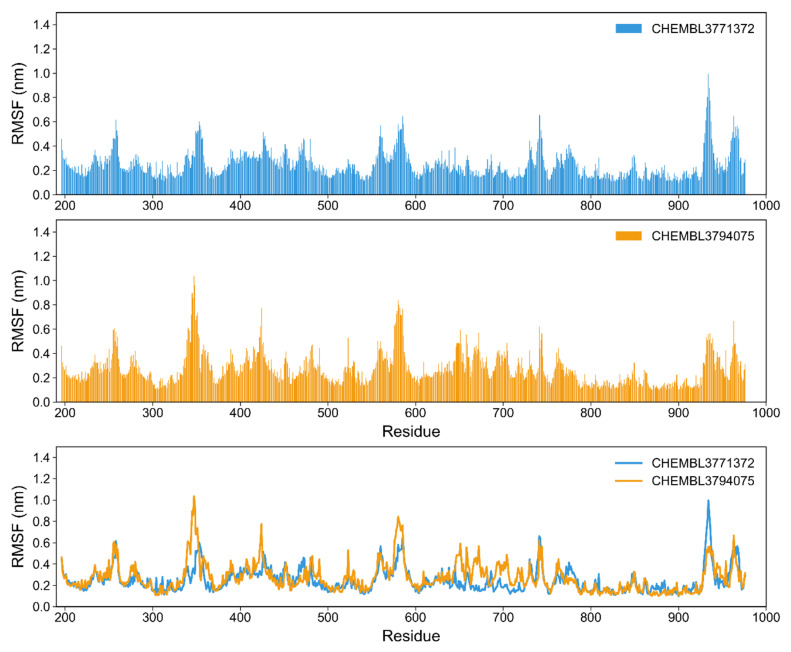
RMSF plots of the EZH2 for the simulations with both selected molecules, CHEMBL3771372 and CHEMBL3794075. Residue IDs in the *x*-axis refer directly to the sequence numbers associated with 5WFC.

**Figure 15:**
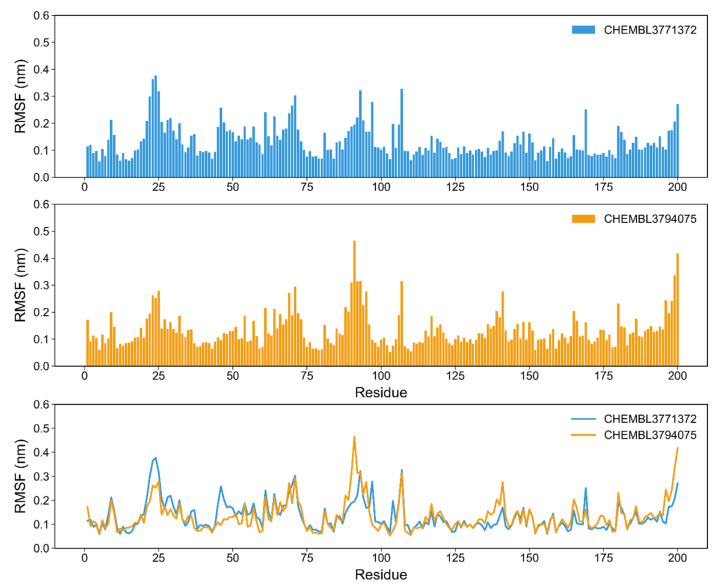
RMSF plots of the P20S’ β5 subunit for the simulations with both selected molecules, CHEMBL3771372 and CHEMBL3794075. Residue IDs in the *x*-axis refer directly to the sequence numbers associated with 5LF3 (chain beta type-5). RMSF values for β5 are shown in Supporting Figure S7.

Assessing the root mean square fluctuation (RMSF) allows monitoring changes in position for the residues within a protein, throughout the simulation. In this work we monitored the fluctuation of the residues in EZH2 (Figure 14) and the residues from the β5 and β6 subunits in P20S (Figure 15). 

In the simulations of the EZH2, the residues that are part of the pocket (301–307 and 804–922) are stable with RMSF < 6 Å (Figure 14). However, in contrast to what was observed for P20S, some segments of the protein reach a RMSF value of 8 Å. Interestingly, both segments with residues from the binding site are flanked on both sides by high RMSF regions. A previous simulation on the human EZH2 SET domain (which coincides with the full binding site in our simulation) shows that the free protein has high RMSF regions such as 688–711 and 744–764 [37]. The last portion is also highly fluctuant in our studies, for both compounds. Beyond this, to our knowledge there are only two MD studies focused on studying the binding to EZH2’s active site, one of which is a research paper [37] and the other is published in conference proceedings [39], and neither discuss fluctuations in residues as a result of ligand presence (either this information is absent altogether, or mobility plots are shown with no information regarding which portion of the residue IDs refers to EZH2).

In P20S all the residues are stable, with RMSF < 6 Å (Figure 15), except for the terminal residues in the beginning of the β6 chain, which are not part of the pocket (Supporting Figure S7). The variation of residues 85–100 shows higher fluctuation for CHEMBL3794075 which partly coincides with residues that make up the binding pocket as identified by SiteFinder in MOE, albeit these overlapping residues correspond to the edge of the pocket. Another segment that shows different fluctuations between compounds corresponds to residues 23–27. In this case, these residues have slightly increased RMSF values for CHEMBL3771372, and part of them is a part of the binding site, in an opposing wall to the prior set of residues, close to THR1. This is in line with the fact that CHEMBL3794075 stays closer to THR1 and, therefore, closer to this second set of residues. The level of fluctuation obtained in this work is significantly smaller than RMSF profiles reported for other inhibitors [52]. 

**Figure 16:**
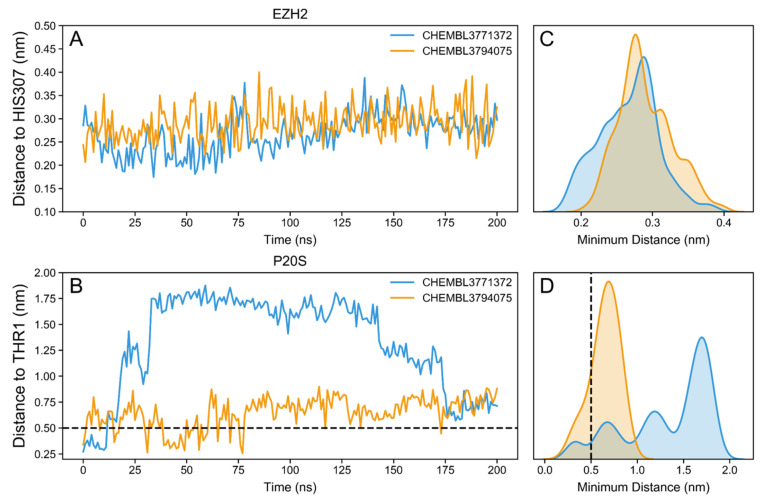
Distance from both docking hits to EZH2’s HIS307 (**A**) and to P20S’s THR1 (**B**). Distribution of minimum distances to HIS307 (**C**) and THR1 (**D**) recorded throughout the full 200 ns simulation.

To assess the particular positioning of the two docking hits with respect to key H-bonding residues, we monitored the shortest distance between each compound and THR1 of the β5 chain of P20S, or HIS307 of EZH2. This revealed that in EZH2 both docking hits remain very close to the reference residue HIS307 (all distances < 4 Å), showing very closely overlapping distance curves, which is very clear from the distribution plot with all distances (Figure 16A,C). This supports the hypothesis that both ligands are promising inhibitors of EZH2. 

Regarding the distance monitoring in P20S, CHEMBL3794075 remains in a much closer distance to P20S’ THR1 throughout the whole simulation, when compared to CHEMB3771372. In fact, while the latter shows only 6.0% of timepoints within 5 Å distance (and all happening at the start of the simulation), the former shows 16.4% of timepoints with this distance. The difference between the distances for the two ligands is also evident from the analysis of the distribution of distance values (Figure 16B,D). Other authors have used this distance as an indicator of the formation of covalent bonds [41] but regardless of whether our compounds are covalent or noncovalent inhibitors, a low distance indicates a higher likelihood of the formation of a stable complex within the P20S pocket. Interestingly, CHEMB3771372 remained outside of the binding pocket for most of the simulation but returned to the β5 binding pocket. However, distance alone is not enough to establish whether the formation of a stable complex is likely to occur, so next we evaluated the H-bonding profile for both ligands. 

**Figure 17:**
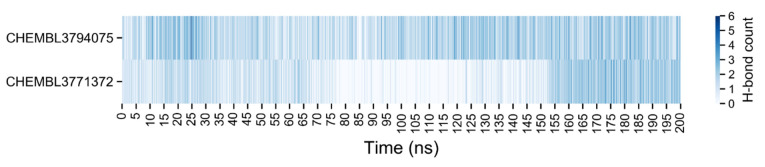
Number of hydrogen bonds between the EZH2 and the ligands, the top from the CHEMBL3771372 and the bottom from the CHEMBL3794075. A detailed plot is shown in Supporting Figures S8 and S9.

**Figure 18:**
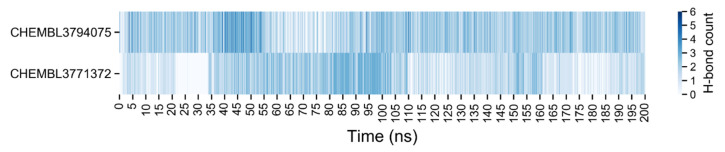
Number of hydrogen bonds between the P20S and the ligands, the top from the CHEMBL3771372 and the bottom from the CHEMBL3794075. A detailed plot is shown in Supporting Figures S10 and S11.

The number of H-bonds between the ligands and the proteins was measured during the simulations for EZH2 and P20S and an overview of the amount of H-bonds as a function of time is shown in Figure 17 and Figure 18, respectively. 

**Figure 19:**
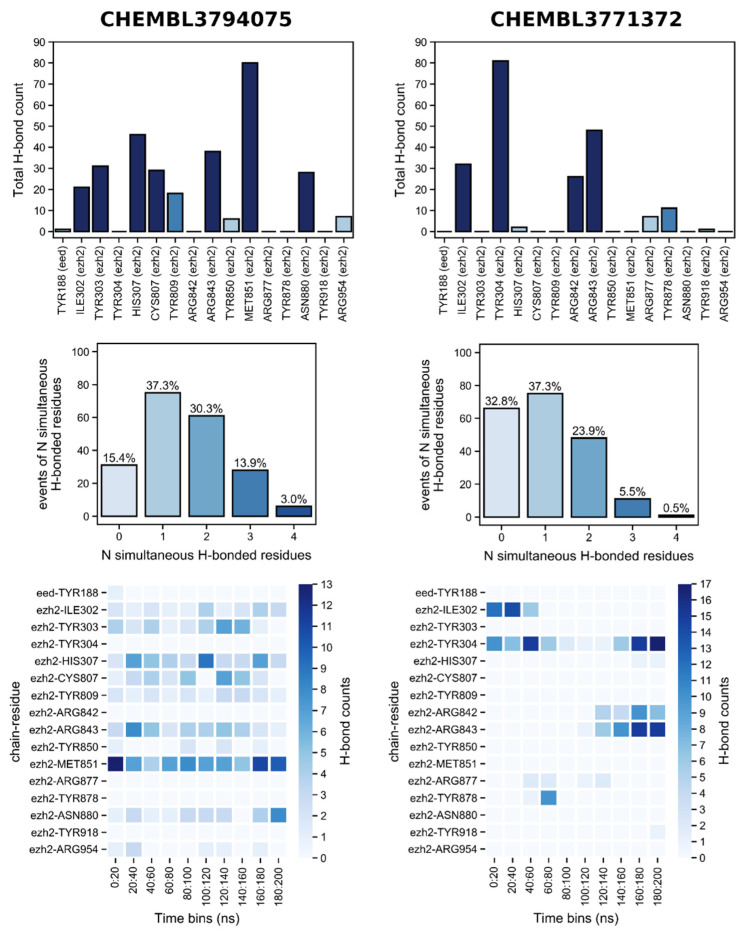
Hydrogen bonds established with EZH2 by the two hit compounds obtained from docking, throughout the 200 ns simulation. For ease of visualisation, the bar plot of total H-bond counts shows higher counts in increasingly darker blue: <10 H-bonds (light blue), 10–20 H-bonds (medium blue), >20 H-bonds (dark blue).

Simulations in EZH2 show that the known P20S inhibitor CHEMBL3794075 surprisingly has a much more promising H-bonding profile than the known EZH2 inhibitor CHEMBL3771372, as evident by the general overview of established H-bonds through time (Figure 19). While the former shows almost continuous interaction with MET851 throughout the full simulation time (adding up to a total of 80 out of 200 ns where this residue is H-bonded), and frequent recurring H-bonding with other residues also throughout almost the entirety of the simulation (ILE302, TYR303, HIS307, TYR809, ARG843, and ASN880). This dramatically contrasts with CHEM3771372 which shows a similar pattern of interaction with only TYR304 and shows more frequent H-bonding with ARG842 and ARG843 for only the last half of the simulation. 

Furthermore, CHEMBL3794075 forms H-bonds with at least one residue much more often than CHEMBL3771372 (84.6 versus 67.2% of time points, respectively), and also establishes H-bonds with two or more residues simultaneously more frequently (75.2 versus 29.9% of time points, respectively). As mentioned earlier there are only two MD studies on EZH2 which can be comparable to ours [37,39] (i.e., which studied the same binding site); however, they do not discuss the H-bonding profile produced in their calculations.

**Figure 20:**
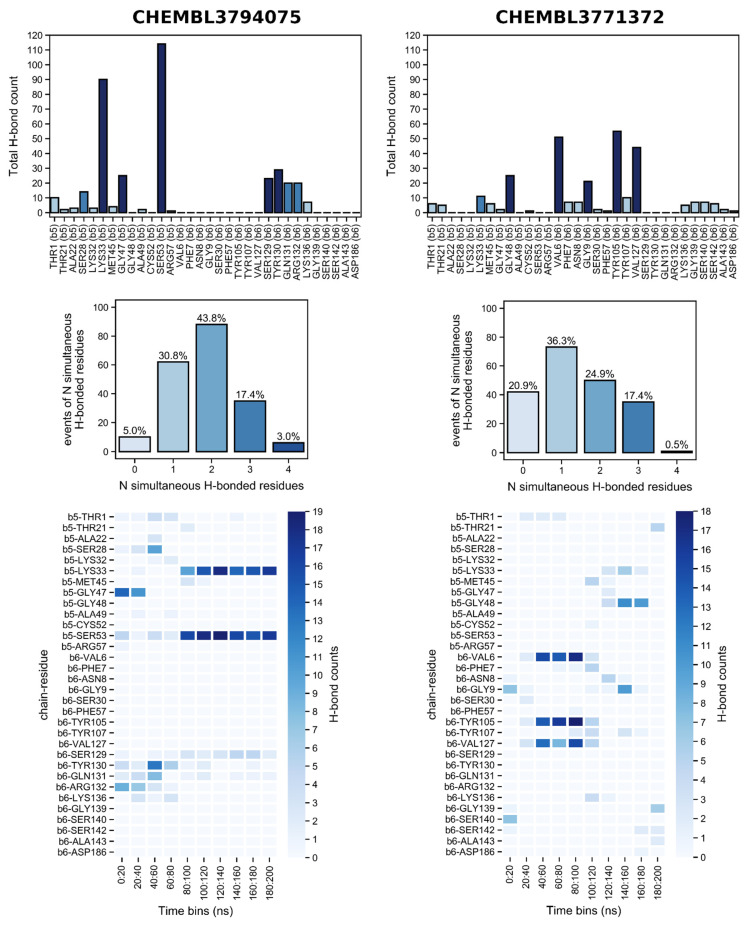
Hydrogen bonds established with P20S by the two hit compounds obtained from docking, throughout the 200 ns simulation. For ease of visualisation, the bar plot of total H-bond counts shows higher counts in increasingly darker blue: <10 H-bonds (light blue), 10–20 H-bonds (medium blue), >20 H-bonds (dark blue).

Simulations in P20S show a very different picture than in EZH2. CHEMBL3794075 is the known P20S inhibitor, so it came as no surprise that it showed many more events of H-bonding throughout the 200 ns simulation when compared to CHEM3771372, with only 5% of timepoints showing no H-bonding for the former versus 20.9% for the latter (Figure 20). Additionally, and perhaps more importantly, simultaneous H-bonds with two or more residues occur in the vast majority of the simulation for CHEMBL3794075, and much more frequently than for CHEMBL3771372 (64.2 versus 42.8% of the time points). Furthermore, LYS33 and SER53 (both in β5) are heavily engaged in H-bonding with CHEMBL3794075 from the 80 ns time point onwards, which contrasts the H-bonding pattern observed for CHEM3771372 in which VAL6, TYR105, and VAL127 (all in β6) are simultaneously H-bonded to the compound for a much briefer period of time, closer to the initial portion of the simulation (40–100 ns). The fact that the second half of the simulation is characterised by much sparser H-bonding is a discouraging sign that indicates CHEM3771372 might not efficiently bind to P20S 20S’ β5 pocket.

Other MD studies on P20S highlight β6 ASP114 as a key H-bonding residue for an inhibitor, but we did not detect this interaction with either hit compound. THR21 was identified in this work; however, it was associated with very sparse contacts, which has also been reported in previous works. Zhang et al. (2009) define it as a weak H-bonding residue owing to the low occurrence of bonding events for THR21 [53]. Additionally, LYS33 and TYR168 were reported to form H-bonds in MD simulations, and many other sparse (i.e., low occupancy) interactions to binding molecules have been identified, namely ASP114 and SER118 (β6) as well as ALA22, GLY23, TRP25, ARG165, and ASN208 (β5) [52]. Among these, we have observed contacts with ALA22 only, which was exclusively detected for CHEMBL3794075. Simultaneous H-bond engagement of THR21, GLY47, and ALA49 with a small molecule has been associated with the stabilisation of strands S2 and S4, with THR21 and ALA49 being also gatekeepers of the binding pocket [53] and, in our case, CHEMBL3794075 shows a considerable engagement with THR21 at the start of the simulation (0–40 ns).

Considering the results produced by the two simulations, the evidence points to CHEMBL3794075 appearing as an overall promising candidate for dual targeting of EZH2+P20S, and potentially more promising than CHEMBL3771372. 

## 3. Conclusions

MM treatment remains very limited despite major breakthroughs in the last 20 years with one of the main issues that hampers the efficacy of current therapies being the development of resistance to treatment. Therefore, polypharmacology therapies, particularly multitargeting inhibitors, have started to be pursued recently; however, there is one promising strategy that remains unexplored—dual targeting of Proteasome 20S (P20S) and EZH2. The reason this strategy is promising stems from experimental findings that have reported that inhibiting EZH2 sensitises P20S for the action of inhibitors. This realisation prompted us to explore a new drug design strategy to find dual EZH2–P20S inhibitors.

In this work, two dual-target inhibitors (EZH2 and P20S proteins) were identified using a combination of computational approaches, namely molecular docking, machine learning, and molecular dynamics, to screen two bioactivity datasets retrieved from ChEMBL (208 EZH2 inhibitors and noninhibitors, and 530 P20S inhibitors and noninhibitors). To achieve this, our first step was to establish the feasibility of having a single molecule that simultaneously inhibits these two proteins. To do so we compared characteristics of the two binding sites as well as the interactions formed with the corresponding X-ray cocrystallised ligands. From this evaluation we concluded that although the shape, volume, and accessible surface area were slightly different, both EZH2 and P20S X-ray cocrystallised ligands (GSK343 and bortezomib, respectively) had some polar and nonpolar regions that allowed us to anticipate possible similar binding modes in the two pockets. This was confirmed when we cross-docked bortezomib into EZH2 and found it shared some of the same H-bonds with X-ray GSK343, which we knew from the literature to be critical. These promising results motivated us to evaluate whether known inhibitors of EZH2 and P20S might bind simultaneously to the two targets. To that end we carried out a molecular docking simulation using the two ChEMBL datasets, and used the obtained PLIFs to build a docking-based predictive model to screen the datasets. This model was able to predict active compounds with very high precision (>90%) and was based on the similarity of residue contact profiles between each compound and each X-ray ligand. This model was applied to predict EZH2 inhibitors among known P20S inhibitors, and vice versa, which yielded two hits: CHEMBL3771372 and CHEMBL3794075.

To complement this, we also analysed the chemical space overlap between both datasets of inhibitors and generated a QSAR classification (decision tree) model using machine learning, based on a total of 202 interpretable molecular descriptors to predict P20S and EZH2 inhibitors. However, when applied to predict the EZH2 and P20S dual inhibitors, we realised that none of these predicted hits coincided with the two hits proposed by molecular docking. We rationalised this with the fact that there is no overlap between EZH2 and P20S chemical spaces, which means using a model trained in one dataset to predict over the other dataset is associated with over-extrapolation and, as a result, low confidence predictions. 

In a final stage, we decided to further study the two molecular docking hits and ignore any potential QSAR hits due to the very low overlap in chemical space between the two datasets which may lead to model predictions being out of the corresponding applicability domains. We carried out molecular dynamics simulations for CHEMBL3771372 and CHEMBL3794075 on the two proteins, and results show that CHEMBL3794075 is particularly promising as a dual-target (EZH2+P20S) inhibitor candidate, due to its ability to establish frequent H-bonds with a variety of residues. Even though CHEMBL3771372 also exhibits some ability to establish stable H-bonds with some residues, these occur over a much shorter period of the simulation.

Beyond the fact that we have proposed here a new possible dual inhibitor against EZH2 and P20S, this work serves as an in silico framework that can be used as a reference in other drug discovery projects for the development of multitargeting drugs. Here we present the full workflow that a drug discovery project could follow, which spans from how to decide on new targets to extracting bioactivity data and building predictive models to find hits. This workflow has a full integration of three complementary methods that can be used to provide different types of information, and can act synergistically to corroborate each other, or as a warning for potential over-extrapolating conclusions.

## 4. Methods

### 4.1. P20S and EZH2 Ligands Dataset Compilation and Preparation

Two datasets were compiled retrieving all available ligands annotated for P20S and EZH2 proteins, respectively, from the ChEMBL 25 database [54], using the SQLite version. These datasets were curated by removing entries whenever they were associated with an empty “Standard value” field, showed symbols of “>” and “≥” under “Standard relation”, showed units that are not convertible to nM (such as “%”, “/M/s”, “/s”, “10′-3/s”, “10′-4/s”, “hr”, or “micromol/min”), or showed an empty value in the “Standard units” field. Entries with “FC” or “Ratio” in the “Standard type” columns were also deleted. Only data with a “confidence score” ≥8 were accepted. All concentration data were converted to nanomolar (nM). After employing these criteria, the final activity data were exclusively expressed in *IC*_50_ and *K*_i_ readouts. This type of aggregation is typical for purposes of partitioning data into qualitative activity classes. Datasets were then prepared using OpenBabel 2.3.0 [55] to remove duplicates, convert SMILES to SDF, protonate, and obtain 3D structures. From the initial datasets containing 1145 and 1044 entries for P20S and EZH2 ligands, respectively, only 530 and 208 molecules (respectively) remained. Whenever multiple activities were associated with the same compound, the smaller activity value was kept. LigPrep and EpiK utilities from Schrödinger (Schrödinger LLC (New York, NY, USA), v.2019) were used to generate ligand ionisation states between pH 5.0 and 9.0 and tautomers. Finally, stereoisomers of the molecules were generated by sampling all the configurations with the maximum of 32 different variations. The geometries were minimised with the OPLS3e force field [56]. The result of this filtration and preparation step was two screening datasets with 6175 and 7389 different structural and chemical possibilities of the ligands for the P20S and EZH2 proteins, respectively.

### 4.2. Preparation of P20S and EZH2 Protein Structures

X-ray structures of P20S and EZH2 complexes were retrieved from the Protein Data Bank (PDB), 5LF3 and 5WFC, respectively. These 3D structures were stripped from all ligands, salts, ions, and water molecules. Whenever covalent bonds existed between the protein and the ligand (P20S), these were broken, and the respective noncovalent structures were recovered. Schrödinger’s Protein Preparation Wizard tool (Maestro) (Schrödinger LLC, v.2019) was used for this minimisation and preparation step. For P20S, only chains K and L were kept (β5 and β6 subunits). For EZH2, chains A and B were kept. 

### 4.3. Structural Analysis of Inhibitors and Protein Pockets

The EZH2 and P20S inhibitors datasets were characterised (only actives were considered) according to key physicochemical features calculated in RDKit [57], and visualised using Seaborn’s boxplot function. The datasets were compared in terms of overlap in Bemis–Murcko scaffolds (also known as Murcko scaffolds) [58] calculated in RDKit, which were then visualised using matplotlib_ven. The structural similarity between both sets was calculated using RDKit, by determining the highest Tanimoto coefficient over Morgan Fingerprints (1024 bits, radius = 2) between each compound of a given dataset and all compounds in the second dataset. This 1 versus all comparison was done for each dataset to produce two similarity distributions plotted using Seaborn’s kdeplot (bandwidth automatically assigned). The chemical space was visualised using sklearn’s t-SNE employed over Morgan Fingerprints of all active compounds of both datasets. All plots were produced in Jupyter, on a Python 3.7 environment.

### 4.4. Molecular Docking (Validation, Parameters, and Simulations)

Docking studies were carried out using X-ray structures 5LF3 for P20S and 5WFC for EZH2 and Schrödinger’s Glide with extra precision (XP) module (Schrödinger LLC, v.2019). To validate the chosen structures for each of the proteins and Glide XP accuracy, self- and cross-docking calculations were performed and their accuracy was assessed based on the ability to correctly place the ligand within the pocket (RMSD of atoms’ placement). RMSD values were calculated using fcon [59]. A Glide scoring grid around the protein structure was generated using the Receptor Grid Generation platform of Schrodinger’s Glide module (Schrödinger LLC, v.2019). A grid of outer box size of 40 × 40 × 40 Å with inner box size of 10 × 10 × 10 Å was generated. The van der Waals radius scaling factor was set to 1, the partial charge cut-off was set to 0.25, and the ligands’ length to be docked was set to a larger than standard maximum of 30 Å. Docking calculations were performed using the Virtual Screening Workflow tool implemented in Maestro (Schrödinger LLC, v.2019). Docking calculations were split into four phases: first, a docking run using Glide HTVS, which retained all states for each molecule; second, a docking run using Glide SP, which retained all good score states; third, a docking run using Glide XP, which retained only the best score state of each molecule; fourth a post-processing run using Prime MM-GBSA. In all stages the conditions were similar, 1 pose per ligand, keep 100% of the compounds in each phase, particle charge cut-off 0.15 with a scaling factor of 0.80 and perform post docking minimisations of the ligand in each stage. Each phase is more accurate than the previous one, thus taking more time to run the molecules. Glide uses two scoring functions. First, to select between protein–ligand complexes of a given ligand, it uses the Emodel scoring function. After that, to rank-order compounds, it uses the GlideScore function. Then, with the final poses of the ligands Protein Ligand Interaction Fingerprints (PLIFs) were calculated using Schrödinger, in order to better study the contacts between the ligands and the pockets.

### 4.5. Prediction Performance Metrics for Molecular Docking

Docking performance was assessed in terms of the ability to accurately recover active compounds from the ChEMBL datasets. To do so, enrichment curves were built using the docking score and the ligand efficiency for the docked ligands. Enrichment curves based on PLIFs similarity were also used, where PLIF similarity was calculated as the Tanimoto coefficient distance between the profile of residue contacts for each ligand and the X-ray ligand of structures 5LF3 (P20S) and 5WFC (EZH2). The subset of residues used to calculate PLIF similarities was additionally optimised testing the *N* most frequently populated residues, where *N* ranged between 5 and 25, with a step of 5.

### 4.6. Building of the Machine Learning (Decision Tree) Classification Model

A total of 202 interpretable molecular descriptors (2D and i3D) were calculated for the two datasets of 530 and 208 molecules for the P20S and EZH2 proteins, respectively (Section 4.1). The datasets with the ligands were divided in 3 classes, actives with an activity < 1 μM, inactives if the activity > 10 μM, and moderate actives for activities between those two thresholds. Then, the datasets were split into training and test sets (80:20% split). The training set was used to build prediction models and the test set used to assess their efficiency in predicting activities. A decision tree classification model was trained using DecisionTreeClassifier in scikit-learn, where hyperparameters (max depth and min leaf size) were optimised through 10-fold cross-validation. The best set of parameters were then used to fit 5 decision tree models using 5 different sets of misprediction weights (“balanced”, 1:1:1, 1:2:2, 1:2:5, and 1:5:10) whose performance was assessed using the test set (results are shown in Supporting Table S2). The best model was finally selected and employed to screen compounds from the two inhibitors’ datasets (i.e., the model built on EZH2 data was used to predict on the P20S data and vice versa). Model selection was done such that precision in all classes was maximised, and as balanced as possible. Several performance metrics were used to assess and select the best model, including: F1 score, precision score, recall score, and accuracy score, which are defined in the following equations, and which all span from 0 to 1 (where 1 corresponds to maximum performance):

$$\mathrm{precision}=\frac{\mathrm{TP}}{\mathrm{TP}+\mathrm{FP}}$$

$$\mathrm{recall}\;=\frac{\mathrm{TP}}{\mathrm{TP}+\mathrm{FN}}$$

$$F1=\frac{2\times \mathrm{precision}\times \mathrm{recall}}{\mathrm{precision}+\mathrm{recall}}$$

$$\mathrm{accuracy}=\frac{\mathrm{TP}+\mathrm{TN}}{\mathrm{TP}+\mathrm{FN}+\mathrm{TN}+\mathrm{FP}}$$

### 4.7. Topology, Systems Setup, and Molecular Dynamics Simulation Protocols

As a starting point for the molecular dynamics simulations the structures of the protein–ligand systems were taken from the docking simulations. Protein and ligand structures were split and saved in different input files. All the molecular dynamics simulations were carried out using GROMACS 2016.3 [60,61,62]. First, we generated a molecular description of the molecules to be simulated. The topology for the input coordinate files for the proteins were obtained directly from the GROMOS-54A7 force field [63] using GROMACS 2016.3 pdb2gmx module [60,61]. For the ligands, the molecular topologies were generated using the ATB server (version 3.0; the united-atom variant and original structure were selected). For the simulations of the P20S protein only the β-rings were used and two non-natural residues were replaced by natural ones that existed in the residue parameter library, 6V1 was replaced with cysteine and M3L was replaced with lysine. The separate topology and structure files for both protein and ligand are combined into a single set of files to continue with the simulation setup. The topology file thus contains all the physical information about all interactions between the atoms of the protein (bonds, angles, torsion angles, Lennard-Jones interactions, and electrostatic interactions). Now that a topology was generated, the next step was to generate a simulation box into which to place this topology. All the protein–ligand systems were inserted into a dodecahedral box and solvated with water molecules, using GROMACS gmx module and the SPC water model [64] to mimic a physiological environment. The net charge of the simulation systems was neutralised adding counterions to the systems. The EZH2 systems were neutralised with 6 Na^+^ ions and the P20S systems with 16 Na^+^ ions. The systems were initially energy minimised in two parts, using a step integrator and a cg integrator. The step integrator allows the steepest descent of the energy of the system. Followed by the cg integrator for the final energy minimisation of the system. Each part had 1000 steps, or less if the system reached the minimum potential energy earlier. The systems were then equilibrated in a small simulation under periodic boundary conditions in the canonical ensemble (*NVT*) followed by another small simulation, but with the isothermal–isobaric ensemble (NPT) at *P* = 1 bar and *T* = 300 K, both using an integration time step of 2 fs. Before running the equilibration step of the simulations, the position of the ligand was restrained. Both simulations had 1 ns of duration, in the NVT ensemble the V-rescale temperature coupling method [65,66] was used to set the temperature, and in the NPT ensemble the Parrinello–Rahman pressure coupling [67] was applied to set the pressure. 

The simulations ran for a total of 200 ns. Later in the analysis all the parts of the simulation were merged. The integration time step was 2 fs, and the coordinates of the alpha carbons were saved every 10 ps. The coupling methods for temperature and pressure were kept apart from the equilibration simulations.

### 4.8. Analysis of the Molecular Dynamics Simulations

The total energy of the system, the root-mean square fluctuation (RMSF) of the protein residues, the minimal distance between the ligand and a key residue in each pocket, the number of hydrogen bonds, and the calculated interaction energy of the protein+ligand system were all used to evaluate the quality of the simulated interaction between the ligands and the proteins. The total energy of the system during the simulation was calculated using the gmx energy command for the system, and the purpose of this analysis was to find out if the system was well minimised and equilibrated.

The RMSF value was calculated for the protein residues using the gmx rmsf command for each chain of interest, the β5 and β6 subunits in the P20S (chains K and L, respectively) and chain A for EZH2. RMSF is a measure of individual residue flexibility, or how much a particular residue moves during a simulation indicating which amino acids in a protein contribute the most to a molecular motion. The minimal distance between the ligands and a chosen residue, THR1 from the β5 chain in the P20S and HIS307 in the EZH2, was computed using distance function in biopandas; this analysis was important for evaluating if the ligand stayed in the pocket or left during the course of the simulation. The distribution of distances was inspected with kernel density estimation, using kdeplot function in Seaborn; this analysis measures the frequency of distance values during the simulation, and plots the probability of the ligand being at a certain distance during the simulation.

Hydrogen bonds between the ligand and the protein were detected with the gmx hbond command, with three options, num, hbm, and hbn. The num option gives the total number of hydrogen bonds at certain frame of the simulation, hbm gives a matrix of donors and acceptors from the protein and from the ligand with all the hydrogen bonds and at which frame they interact during the simulation, and hbn gives the index file of all donor and acceptor atoms from the protein and the ligand. All the information from the three files was combined using a Python script in order to be analysed and plot the graphics. 

### 4.9. Data Management, Data Analysis Plots and Figures

All raw data was analysed using Jupyter, running on a Python3 environment. For the data assembly and analysis we used pandas as numpy. We used RDKit to handle and draw 2D chemical structures, and Seaborn, Matplotlib, and Matplotlib_venn to produce all plots. The chemical space embedding (i.e., chemical space visualisation) was done using the t-SNE function in scikit-learn. 3D structures were drawn in PyMOL (directly or indirectly through PLIP tools).

## Figures and Tables

**Table 1:** Values of the similarity of the PLIFs between the selected molecules and the X-ray ligand, and the IC_50_ values for the known activities.

	PLIFs Similarity		IC_50_ (nM)	
	EZH2	P20S	EZH2	P20S
CHEMBL3794075	0.900	0.917	unknown	26.24
CHEMBL3771372	0.900	0.917	10.00	unknown

**Table 2:** Performance of the QSAR models built with the P20S inhibitors dataset and the EZH2 inhibitors dataset, respectively, measured on the test set.

	Class	N (Test)	Precision	Recall	F1	Overall Accuracy
**EZH2**	actives	29	0.84	0.90	0.87	0.76
	moderate actives	6	0.60	0.43	0.50	
	inactives	7	0.50	0.50	0.50	
**P20S**	actives	74	0.84	0.93	0.88	0.77
	moderate actives	14	0.44	0.44	0.44	
	inactives	18	0.83	0.36	0.50	

## Data Availability

Any produced data from the different in silico studies is available upon request to the authors.

## Supplementary Materials

The following are available online. Table S1: Pocket residues identified by SiteFinder in MOE2020, Table S2: Performance of the different preliminary machine learning (decision tree models) built with different weight schemes applied to actives (“A”), inactives (“I”) and moderate actives (“M”). Balanced under the misprediction weights corresponds to the “balanced” option in the class_weight parameter of sklearn’s DecisionTreeClassifier. PRE: precision, REC: recall, F1: F1 score, N: number of compounds. The distribution of predictions for every class of EZH2 and P20S datasets are shown in Supporting Tables S3 and S4, Table S3: Number of predicted actives by the EZH2 decision tree model, Table S4: Number of predicted actives by the P20S decision tree model, Figure S1: Enrichment curves for the score, ligand efficiency and PLIFs similarity for P20S and EZH2 datasets, Figure S2: Ligands placed in the EZH2 pocket, where poses are obtained from docking or from X-ray data. (A) Side view of pocket (B) Front view of the pocket. CHEMBL3771372 (magenta), X-ray ligand GSK343 (cyan) and CHEMBL3794075 (yellow), Figure S3: Ligands placed in the EZH2 pocket, where poses are obtained from docking or from X-ray data. (A) Side view of pocket (B) Front view of the pocket. CHEMBL3771372 (magenta), X-ray ligand GSK343 (cyan) and CHEMBL3794075 (yellow), Figure S4: Decision tree model for EZH2 inhibitors, Figure S5: Decision tree model for Proteasome 20S inhibitors, Figure S6: Total energy of the systems, protein with ligand in water, during the course of the simulation, Figure S7: RMSF values for residues in subunit β6 of P20S, Figure S8: Hydrogen bonds established by CHEMBL3794075 with EZH2, Figure S9: Hydrogen bonds established by CHEMBL3771372 with EZH2, Figure S10: Hydrogen bonds established by CHEMBL3794075 with P20S, Figure S11: Hydrogen bonds established by CHEMBL3771372 with P20S.

## Sample Availability

Samples of the compounds are not available from the authors.

## References

[1] Palumbo A., Anderson K. (2011). Multiple myeloma. N. Engl. J. Med..

[2] Branagan A., Lei M., Lou U., Raje N. (2020). Current treatment strategies for multiple myeloma. J. Oncol. Pract..

[3] Raghavendra N.M., Pingili D., Kadasi S., Mettu A., Prasad S.V.U.M. (2018). Dual or multi-targeting inhibitors: The next generation anticancer agents. Eur. J. Med. Chem..

[4] Cavalli A., Bolognesi M.L., Minarini A., Rosini M., Tumiatti V., Recanatini M., Melchiorre C. (2008). Multi-target-directed ligands to combat neurodegenerative diseases. J. Med. Chem..

[5] East S.P., Silver L.L. (2012). Multitarget ligands in antibacterial research: Progress and opportunities. Expert Opin. Drug Discov..

[6] Tomaselli D., Lucidi A., Rotili D., Mai A. (2020). Epigenetic polypharmacology: A new frontier for epi-drug discovery. Med. Res. Rev..

[7] Alcaro S., Bolognesi M.L., García-Sosa A.T., Rapposelli S. (2019). Editorial: Multi-target-directed ligands (MTDL) as challenging research tools in drug discovery: From design to pharmacological evaluation. Front. Chem..

[8] Guedes R.A., Aniceto N., Andrade M.A.P., Salvador J.A.R., Guedes R.C. (2019). Chemical patterns of proteasome inhibitors: Lessons learned from two decades of drug design. Int. J. Mol. Sci..

[9] Crawford L.J., Irvine A.E. (2013). Targeting the ubiquitin proteasome system in haematological malignancies. Blood Rev..

[10] Narayanan S., Cai C.-Y., Assaraf Y.G., Guo H.-Q., Cui Q., Wei L., Huang J.-J., Ashby C.R., Chen Z.-S. (2019). Targeting the ubiquitin-proteasome pathway to overcome anti-cancer drug resistance. Drug Resist. Updat..

[11] Fricker L.D. (2020). Proteasome inhibitor drugs. Annu. Rev. Pharmacol. Toxicol..

[12] Gandolfi S., Laubach J.P., Hideshima T., Chauhan D., Anderson K.C., Richardson P.G. (2017). The proteasome and proteasome inhibitors in multiple myeloma. Cancer Metastasis Rev..

[13] Chapman M.A., Lawrence M.S., Keats J., Cibulskis K., Sougnez C., Schinzel A.C., Harview C., Brunet J.-P., Ahmann G.J., Adli M. (2011). Initial genome sequencing and analysis of multiple myeloma. Nature.

[14] Walker B.A., Wardell C., Chiecchio L., Smith E.M., Boyd K., Neri A., Davies F.E., Ross F.M., Morgan G. (2011). Aberrant global methylation patterns affect the molecular pathogenesis and prognosis of multiple myeloma. Blood.

[15] Pawlyn C., Bright M., Buros A.F., Stein C.K., Walters Z., Aronson L., Mirabella F., Jones J.R., Kaiser M.F., Walker B. (2017). Overexpression of EZH2 in multiple myeloma is associated with poor prognosis and dysregulation of cell cycle control. Blood Cancer J..

[16] Pawlyn C., Morgan G.J. (2017). Evolutionary biology of high-risk multiple myeloma. Nat. Rev. Cancer.

[17] Chapman-Rothe N., Curry E., Zeller C., Liber D., Stronach E., Gabra H., Ghaem-Maghami S., Brown R. (2013). Chromatin H3K27me3/H3K4me3 histone marks define gene sets in high-grade serous ovarian cancer that distinguish malignant, tumour-sustaining and chemo-resistant ovarian tumour cells. Oncogene.

[18] Yu J., Yu J., Rhodes D.R., Tomlins S.A., Cao X., Chen G., Mehra R., Wang X., Ghosh D., Shah R.B. (2007). A polycomb repression signature in metastatic prostate cancer predicts cancer outcome. Cancer Res..

[19] Suvà M.-L., Riggi N., Janiszewska M., Radovanovic I., Provero P., Stehle J.-C., Baumer K., Le Bitoux M.-A., Marino D., Cironi L. (2009). EZH_2_ is essential for glioblastoma cancer stem cell maintenance. Cancer Res..

[20] Dubuc A.M., Remke M., Korshunov A., Northcott P.A., Zhan S.H., Mendez-Lago M., Kool M., Jones D.T.W., Unterberger A., Morrissy A.S. (2013). Aberrant patterns of H3K4 and H3K27 histone lysine methylation occur across subgroups in medulloblastoma. Acta Neuropathol..

[21] Sneeringer C.J., Scott M.P., Kuntz K.W., Knutson S.K., Pollock R.M., Richon V.M., Copeland R.A. (2010). Coordinated activities of wild-type plus mutant EZH2 drive tumor-associated hypertrimethylation of lysine 27 on histone H3 (H3K27) in human B-cell lymphomas. Proc. Natl. Acad. Sci. USA.

[22] Rizq O., Mimura N., Oshima M., Saraya A., Koide S., Kato Y., Aoyama K., Nakajima-Takagi Y., Wang C., Chiba T. (2017). Dual inhibition of EZH2 and EZH1 sensitizes PRC2-dependent tumors to proteasome inhibition. Clin. Cancer Res..

[23] Ashtiani M., Salehzadeh-Yazdi A., Razaghi-Moghadam Z., Hennig H., Wolkenhauer O., Mirzaie M., Jafari M. (2018). A systematic survey of centrality measures for protein-protein interaction networks. BMC Syst. Biol..

[24] Peters J.U. (2013). Polypharmacology—Foe or friend?. J. Med. Chem..

[25] Albertini C., Naldi M., Petralla S., Strocchi S., Grifoni D., Monti B., Bartolini M., Bolognesi M. (2021). From combinations to single-molecule polypharmacology—cromolyn-ibuprofen conjugates for alzheimer’s disease. Molecules.

[26] Ramsay R.R., Popovic-Nikolicb M.R., Nikolic K., Uliassi E., Bolognesi M.L. (2018). A perspective on multi-target drug discovery and design for complex diseases. Clin. Transl. Med..

[27] Ravikumar B., Aittokallio T. (2018). Improving the efficacy-safety balance of polypharmacology in multi-target drug discovery. Expert Opin. Drug Discov..

[28] Bhatia S., Krieger V., Groll M., Osko J., Reßing N., Ahlert H., Borkhardt A., Kurz T., Christianson D.W., Hauer J. (2018). Discovery of the first-in-class dual histone deacetylase-proteasome inhibitor. J. Med. Chem..

[29] Zhou Y., Liu X., Xue J., Liu L., Liang T., Li W., Yang X., Hou X., Fang H. (2020). Discovery of peptide boronate derivatives as histone deacetylase and proteasome dual inhibitors for overcoming bortezomib resistance of multiple myeloma. J. Med. Chem..

[30] Zhu M., Harshbarger W.D., Robles O., Krysiak J., Hull K.G., Cho S.W., Richardson R.D., Yang Y., Garcia A., Spiegelman L. (2017). A strategy for dual inhibition of the proteasome and fatty acid synthase with belactosin C-orlistat hybrids. Bioorg. Med. Chem..

[31] Bratkowski M., Yang X., Liu X. (2018). An evolutionarily conserved structural platform for PRC2 inhibition by a class of Ezh2 inhibitors. Sci. Rep..

[32] Schrader J., Heinneberg F., Mata R.A., Tittmann K., Schneider T.R., Stark H., Bourenkow G., Chari A. (2016). The inhibition mechanism of human 20S proteasomes enables next-generation inhibitor design. Science.

[33] Lyu J., Wang S., Balius T., Singh I., Levit A., Moroz Y., O’Meara M.J., Che T., Algaa E., Tolmachova K. (2019). Ultra-large library docking for discovering new chemotypes. Nature.

[34] Zhou Q., Jia L., Du F., Dong X., Sun W., Wang L., Chen G. (2020). Design, synthesis and biological activities of pyrrole-3-carboxamide derivatives as EZH2 (enhancer of zeste homologue 2) inhibitors and anticancer agents. N. J. Chem..

[35] Huang K., Sun R., Chen J., Yang Q., Wang Y., Zhang Y., Xie K., Zhang T., Li R., Zhao Q. (2020). A novel EZH2 inhibitor induces synthetic lethality and apoptosis in PBRM1-deficient cancer cells. Cell Cycle.

[36] Yang X., Li F., Konze K.D., Meslamani J., Ma A., Brown P.J., Zhou M.-M., Arrowsmith C.H., Kaniskan H., Vedadi M. (2016). Structure-activity relationship studies for enhancer of zeste homologue 2 (EZH2) and enhancer of zeste homologue 1 (EZH1) inhibitors. J. Med. Chem..

[37] Aier I., Varadwaj P., Raj U. (2016). Structural insights into conformational stability of both wild-type and mutant EZH2 receptor. Sci. Rep..

[38] Zhu K., Du D., Yang R., Tao H., Zhang H. (2020). Identification and assessments of novel and potent small-molecule inhibitors of EED–EZH2 interaction of polycomb repressive complex 2 by computational methods and biological evaluations. Chem. Pharm. Bull..

[39] Aier I., Raj U. Exploring the role of EZH2 (PRC2) as epigenetic target. Proceedings of the 2016 International Conference on Bioinformatics and Systems Biology (BSB).

[40] Raj U., Kumar H., Gupta S., Varadwaj P. (2016). Identification of Novel Inhibitors for Disrupting EZH2-EED Interactions Involved in Cancer Epigenetics: An In-Silico Approach. Curr. Proteom..

[41] Li A., Sun H., Du L., Wu X., Cao J., You Q., Li Y. (2014). Discovery of novel covalent proteasome inhibitors through a combination of pharmacophore screening, covalent docking, and molecular dynamics simulations. J. Mol. Model..

[42] Di Giovanni C., Ettari R., Sarno S., Rotondo A., Bitto A., Squadrito F., Altavilla D., Schirmeister T., Novellino E., Grasso S. (2016). Identification of noncovalent proteasome inhibitors with high selectivity for chymotrypsin-like activity by a multistep structure-based virtual screening. Eur. J. Med. Chem..

[43] Yadav D., Mishra B.N., Khan F. (2020). Quantitative structure-activity relationship and molecular docking studies on human proteasome inhibitors for anticancer activity targeting NF-κB signaling pathway. J. Biomol. Struct. Dyn..

[44] Kazi A., Lawrence H., Guida W.C., McLaughlin M.L., Springett G.M., Berndt N., Yip R.M.L., Sebti S.M. (2009). Discovery of a novel proteasome inhibitor selective for cancer cells over non-transformed cells. Cell Cycle.

[45] Arba M., Nur-Hidayat A., Surantaadmaja S.I., Tjahjono D.H. (2018). Pharmacophore-based virtual screening for identifying β5 subunit inhibitor of 20S proteasome. Comput. Biol. Chem..

[46] Uysal S., Soyer Z., Saylam M., Tarikogullari A.H., Yilmaz S., Kirmizibayrak P.B. (2021). Design, synthesis and biological evaluation of novel naphthoquinone-4-aminobenzensulfonamide/carboxamide derivatives as proteasome inhibitors. Eur. J. Med. Chem..

[47] Adasme M.F., Linnemann K.L., Bolz S.N., Kaiser F., Salentin S., Haupt V.J., Schroeder M. (2021). PLIP 2021: Expanding the scope of the protein-ligand interaction profiler to DNA and RNA. Nucleic Acids Res..

[48] Subramaniam S., Mehrotra M., Gupta D. (2011). Support vector machine based prediction of P. falciparum proteasome inhibitors and development of focused library by molecular docking. Comb. Chem. High Throughput Screen..

[49] Sánchez-Cruz N., Medina-Franco J.L. (2021). Epigenetic target profiler: A web server to predict epigenetic targets of small molecules. J. Chem. Inf. Model..

[50] Cruciani G., Crivori P., Carrupt P.A., Testa B. (2000). Molecular fields in quantitative structure-permeation relationships: The VolSurf approach. J. Mol. Struct. THEOCHEM.

[51] Moorthy N.S.H.N., Ramos M.J., Fernandes P.A. (2012). Structural analysis of structurally diverse α-glucosidase inhibitors for active site feature analysis. J. Enzym. Inhib. Med. Chem..

[52] Arba M., Nur-Hidayat A., Ruslin, Yusuf M., Sumarlin, Hertadi R., Wahyudi S.T., Surantaadmaja S.I., Tjahjono D.H. (2018). Molecular modeling on porphyrin derivatives as β5 subunit inhibitor of 20S proteasome. Comput. Biol. Chem..

[53] Zhang S., Shi Y., Jin H., Liu Z., Zhang L., Zhang L. (2009). Covalent complexes of proteasome model with peptide aldehyde inhibitors MG132 and MG101: Docking and molecular dynamics study. J. Mol. Model..

[54] Gaulton A., Hersey A., Nowotka M., Bento A.P.S.F.F., Chambers J., Mendez D., Mutowo P., Atkinson F., Bellis L., Uhalte E.C. (2017). The ChEMBL database in 2017. Nucleic Acids Res..

[55] O’Boyle N.M., Banck M., A James C., Morley C., Vandermeersch T., Hutchison G. (2011). Open Babel: An open chemical toolbox. J. Cheminform..

[56] Roos K., Wu C., Damm W., Reboul M., Stevenson J.M., Lu C., Dahlgren M.K., Mondal S., Chen W., Wang L. (2019). OPLS3e: Extending Force Field Coverage for Drug-Like Small Molecules. J. Chem. Theory Comput..

[57] RDKit: Open-Source Cheminformatics. http://www.rdkit.org.

[58] Bemis G.W., Murcko M.A. (1996). The properties of known drugs. 1. Molecular frameworks. J. Med. Chem..

[59] Neudert G., Klebe G. (2011). fconv: Format conversion, manipulation and feature computation of molecular data. Bioinformatics.

[60] Van Der Spoel D., Lindahl E., Hess B., Groenhof G., Mark A.E., Berendsen H.J. (2005). GROMACS: Fast, flexible, and free. J. Comput. Chem..

[61] Lindahl E., Hess B., Van Der Spoel D. (2001). GROMACS 3.0: A package for molecular simulation and trajectory analysis. J. Mol. Model..

[62] Hess B., Kutzner C., Van Der Spoel D., Lindahl E. (2008). GROMACS 4: Algorithms for highly efficient, load-balanced, and scalable molecular simulation. J. Chem. Theory Comput..

[63] Huang W., Lin Z., Van Gunsteren W.F. (2011). Validation of the GROMOS 54A7 force field with respect to β-peptide folding. J. Chem. Theory Comput..

[64] Berendsen H.J.C., Grigera J.R., Straatsma T.P. (1987). The missing term in effective pair potentials. J. Phys. Chem..

[65] Berendsen H.J.C., Postma J.P.M., Van Gunsteren W.F., Di Nola A., Haak J.R. (1984). Molecular dynamics with coupling to an external bath. J. Chem. Phys..

[66] Bussi G., Donadio D., Parrinello M. (2007). Canonical sampling through velocity rescaling. J. Chem. Phys..

[67] Parrinello M., Rahman A. (1981). Polymorphic transitions in single crystals: A new molecular dynamics method. J. Appl. Phys..

