# Supplementary material for: Exploring EZH2-Proteasome Dual-Targeting Drug Discovery through a Computational Strategy to Fight Multiple Myeloma
Source: Molecules. 2021 Sep 14;26(18):5574. doi: 10.3390/molecules26185574 (PMC8468724; doi:10.3390/molecules26185574)
Supplement: Supplementary file 1 [file molecules-26-05574-s001.zip › molecules-1332387-supplementary.pdf]

Supporting Information for "*Exploring EZH2-Proteasome Dual-Targeting Drug Discovery through a Computational Strategy to Fight Multiple Myeloma*"

Filipe Estrada, Silvia Miccoli, Natália Aniceto, Alfonso T. Garcia-Sosa, Rita C. Guedes

SUPPORTING TABLES

Supporting Table S1. Pocket residues identified by SiteFinder in MOE2020.

Proteins (PDB ID)	Chains	Pocket residues
5LF3 (P20S)	***chain K (β5)***	THR1 ARG19 ALA20 THR21 TYR25 ALA27 SER28 THR30 VAL31 LYS32 LYS33 ILE35 MET45 ALA46 GLY47 GLY48 ALA49 ALA50 SER53 PHE54 GLU56 ARG57 SER96 MET97 GLY98 GLY129 SER130 TYR169
	chain L (β6)	SER93 TYR97 TYR107 ASN108 SER123 PHE124 ASP125 PRO126 GLY128 SER129 TYR130 GLN131 LYS136
5WFC (EZH2)	***chain A (EED)***	ASP159 THR161 SER187 TYR188 ASP189
	chain B (EZH2)	PRO301 ILE302 TYR303 TYR304 PHE305 HIS307 LEU804 ALA806 CYS807 GLY808 TYR809 TYR826 ARG842 ARG843 TYR850 MET851 CYS852 SER854 PHE855 LEU856 VAL867 ASP868 ALA869 ALA870 SER876 ARG877 TYR878 ILE879 ASN880 HIS881 PHE916 TYR918 PHE922

Supporting Table S2: Performance of the different preliminary machine learning (decision tree models) built with different weight schemes applied to actives ("A"), inactives ("I") and moderate actives ("M"). Balanced under the misprediction weights corresponds to the "balanced" option in the *class_weight* parameter of sklearn's *DecisionTreeClassifier*. PRE: precision, REC: recall, F1: F1 score, N: number of compounds. The distribution of predictions for every class of EZH2 and P20S datasets are shown in Supporting Table S3 and S4.

		**P20S**				**EZH2**			
misprediction weights (A : M : I)		**PRE**	**REC**	**F1**	**N**	**PRE**	**REC**	**F1**	**N**
1 : 1 : 1	active	**0.84**	0.93	0.88	74	**0.84**	0.90	0.87	29
	medium	**0.44**	0.44	0.44	18	**0.60**	0.43	0.50	7
	inactive	**0.83**	0.36	0.50	14	**0.50**	0.50	0.50	6
	accuracy	0.77			106	0.76			42
1 : 2 : 2	active	0.88	0.92	0.90	74	0.81	0.90	0.85	29
	medium	0.53	0.50	0.51	18	0.00	0.00	0.00	7
	inactive	0.67	0.57	0.62	14	0.50	0.83	0.62	6
	accuracy	0.80			106	0.74			42

1 : 2 : 5	active	0.89	0.89	0.89	74	0.81	0.90	0.85	29
	medium	0.38	0.33	0.35	18	0.00	0.00	0.00	7
	inactive	0.31	0.36	0.33	14	0.50	0.83	0.62	6
	accuracy	0.73			106	0.74			42
1 : 5 : 10	active	0.89	0.91	0.90	74	0.83	0.52	0.64	29
	medium	0.50	0.33	0.40	18	0.24	0.57	0.33	7
	inactive	0.42	0.57	0.48	14	0.43	0.50	0.46	6
	accuracy	0.76			106	0.52			42
Balanced	active	0.87	0.84	0.86	74	0.81	0.90	0.85	29
	medium	0.39	0.39	0.39	18	0.00	0.00	0.00	7
	inactive	0.53	0.64	0.58	14	0.50	0.83	0.62	6
	accuracy	0.74			106	0.74			42

Supporting Table S3. Number of predicted actives by the EZH2 decision tree model.

P20S class	EZH2 prediction	N
active	active	260
	moderate	9
	inactive	104
moderate	active	62
	moderate	19
	inactive	20
inactive	active	28
	moderate	12
	inactive	13

Supporting Table S4. Number of predicted actives by the P20S decision tree model.

EZH2 class	P20S prediction	N
active	active	12
	moderate	115
	inactive	11
moderate	active	11
	moderate	22
	inactive	5
inactive	active	5
	moderate	20
	inactive	7

SUPPORTING FIGURES

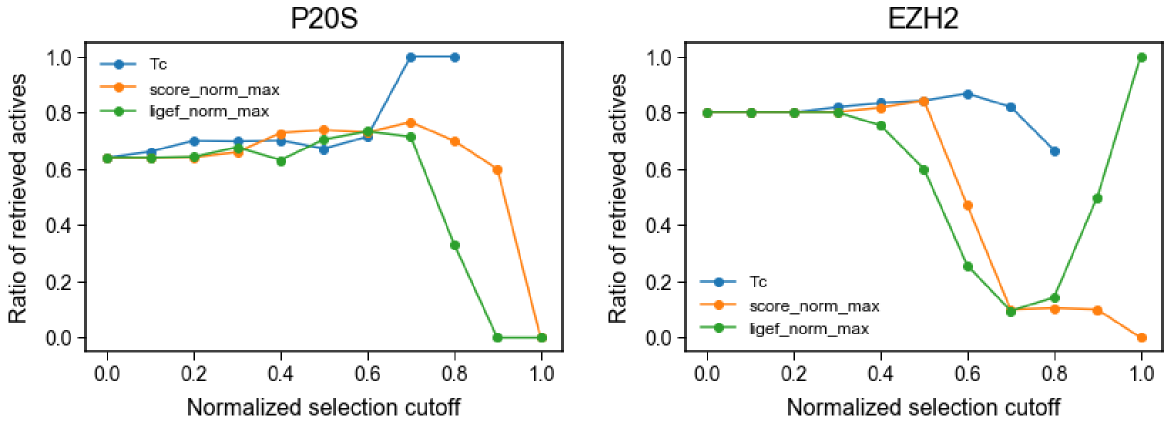

Supporting Figure S1. Enrichment curves for the score, ligand efficiency and PLIFs similarity for P20S and EZH2 datasets.

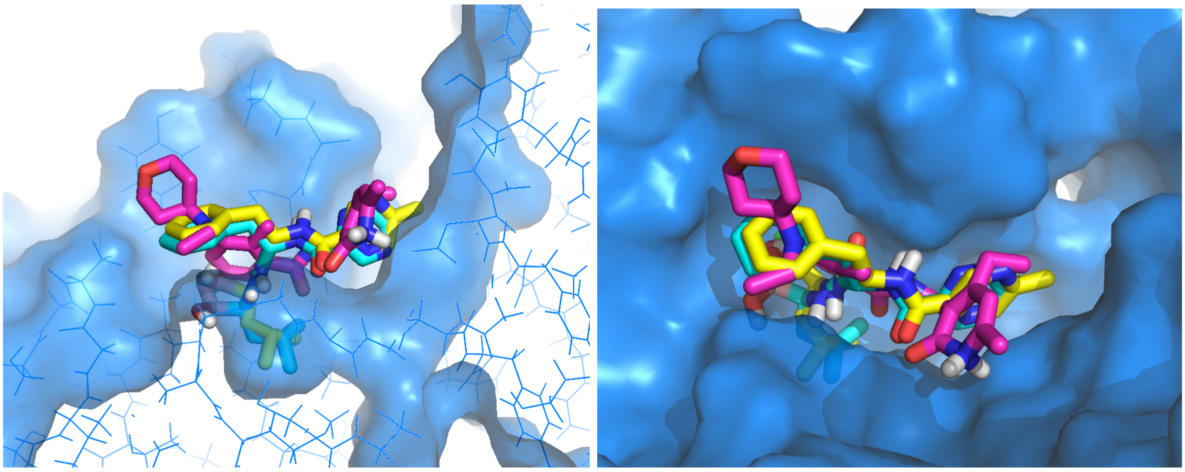

Supporting Figure S2. Ligands placed in the EZH2 pocket, where poses are obtained from docking or from X-ray data. (A) Side view of pocket (B) Front view of the pocket. CHEMBL3771372 (magenta), X-ray ligand GSK343 (cyan) and CHEMBL3794075 (yellow).

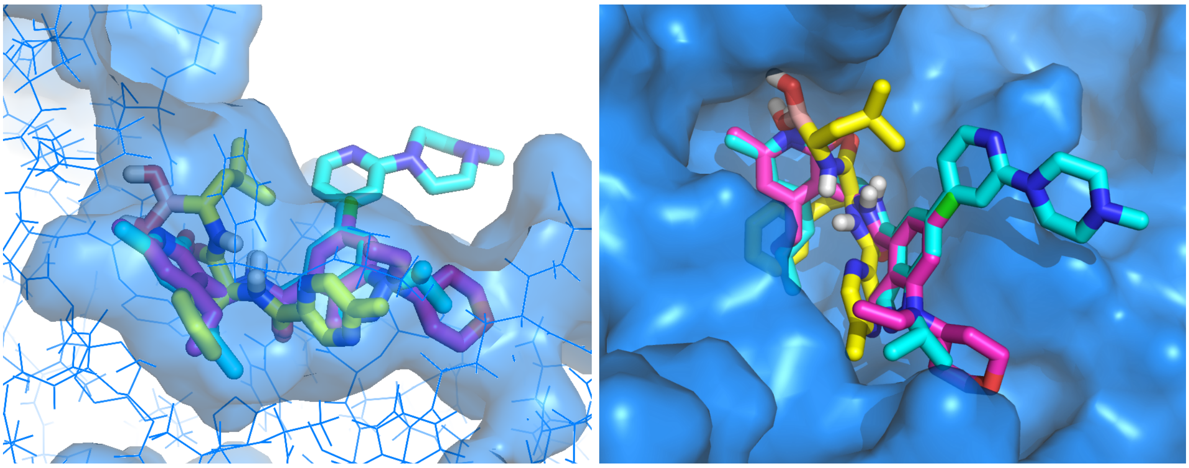

Supporting Figure S3. Ligands placed in the EZH2 pocket, where poses are obtained from docking or from X-ray data. (A) Side view of pocket (B) Front view of the pocket. CHEMBL3771372 (magenta), X-ray ligand GSK343 (cyan) and CHEMBL3794075 (yellow).

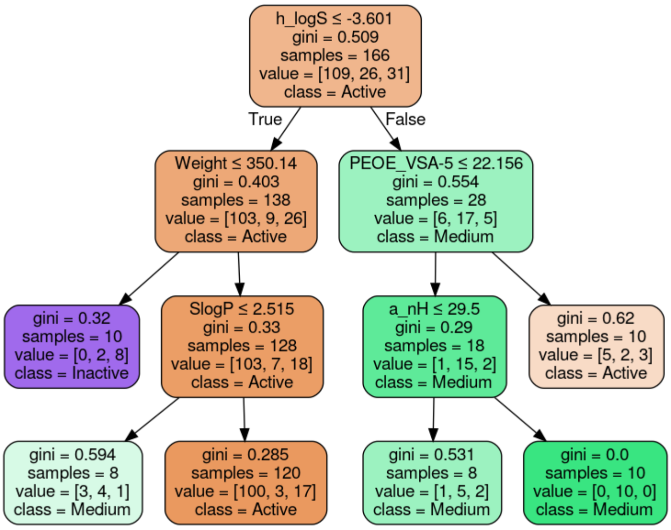

Supporting Figure S4. Decision tree model for EZH2 inhibitors.

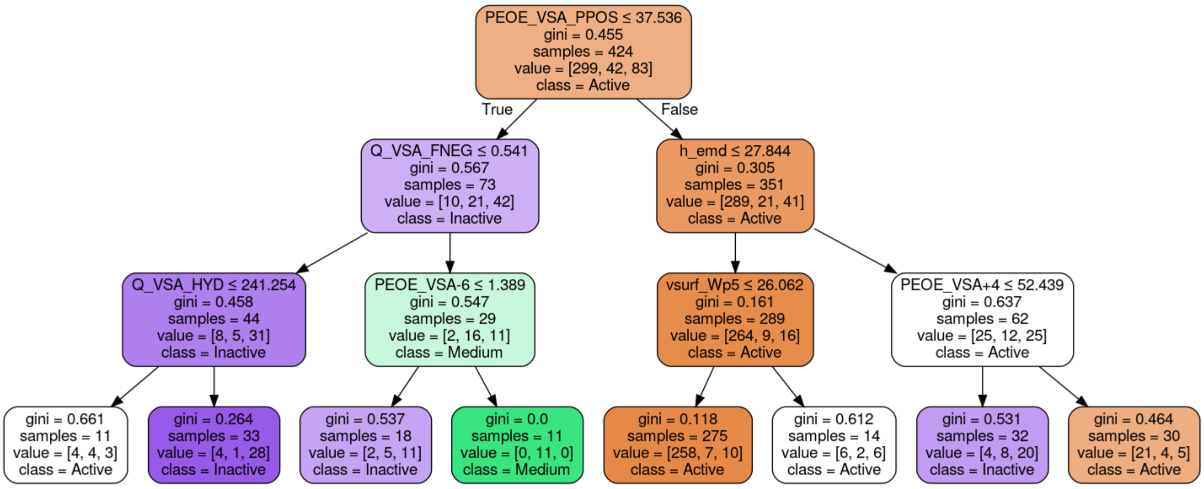

Supporting Figure S5. Decision tree model for Proteasome 20S inhibitors.

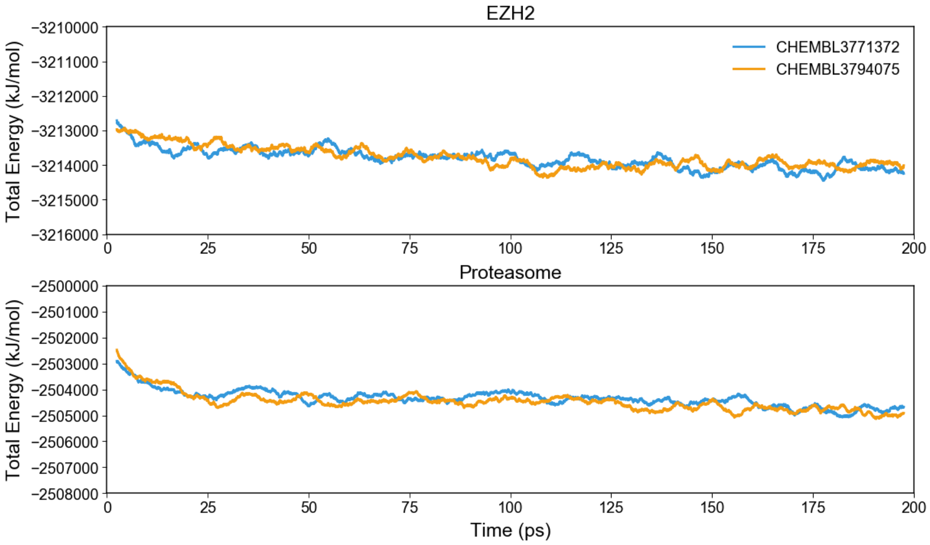

Supporting Figure S6. Total energy of the systems, protein with ligand in water, during the course of the simulation.

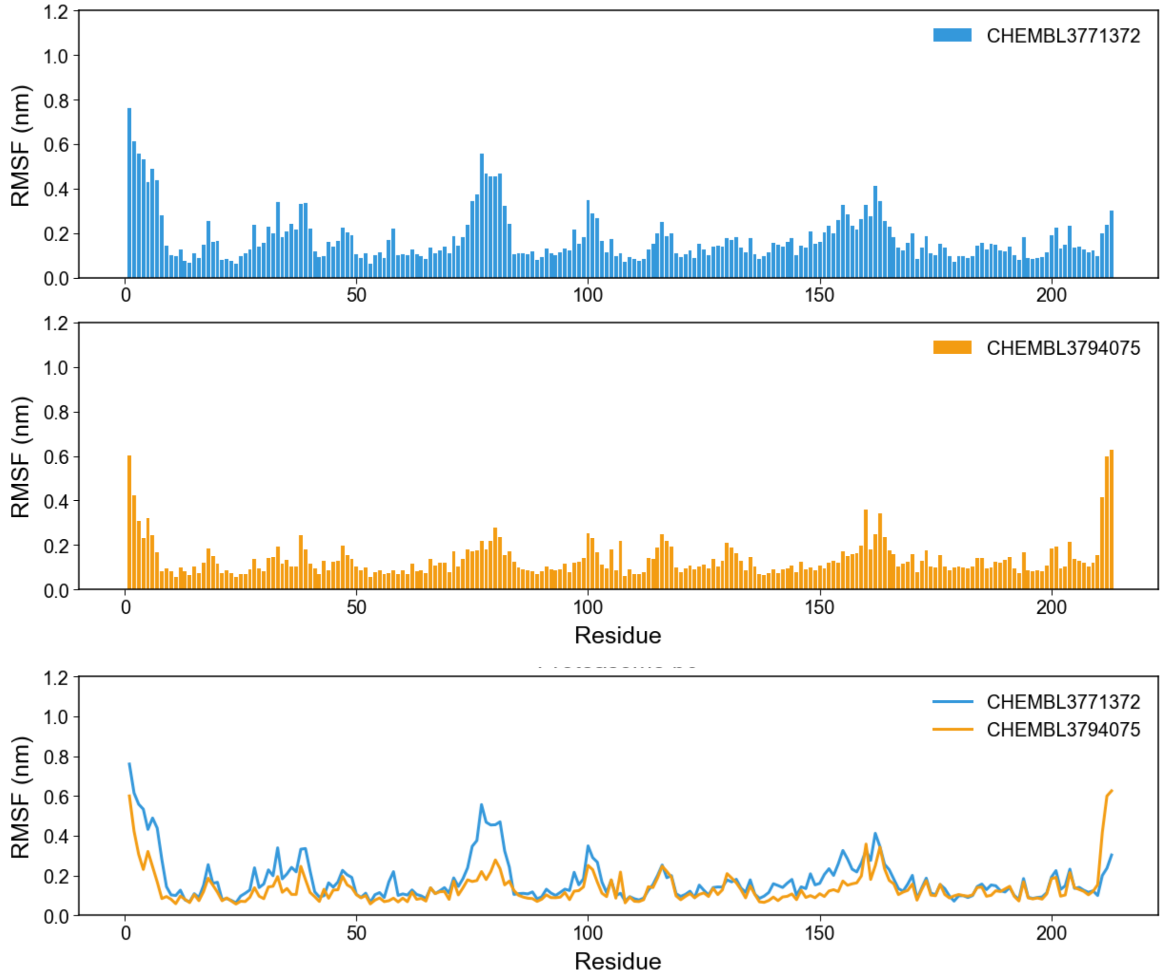

Supporting Figure S7. RMSF values for residues in subunit β6 of P20S.

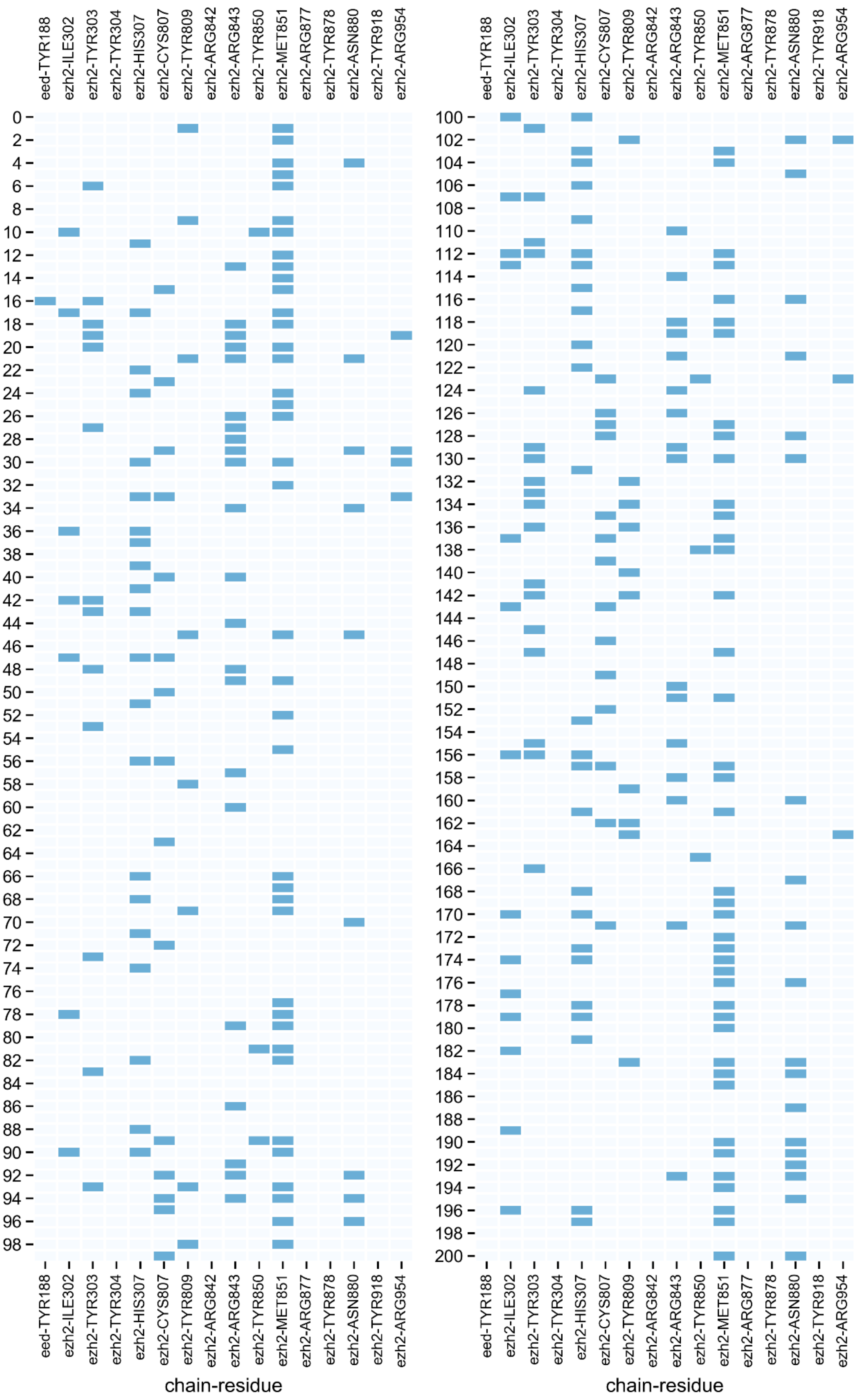

Supporting Figure S8. Hydrogen bonds established by CHEMBL3794075 with EZH2.

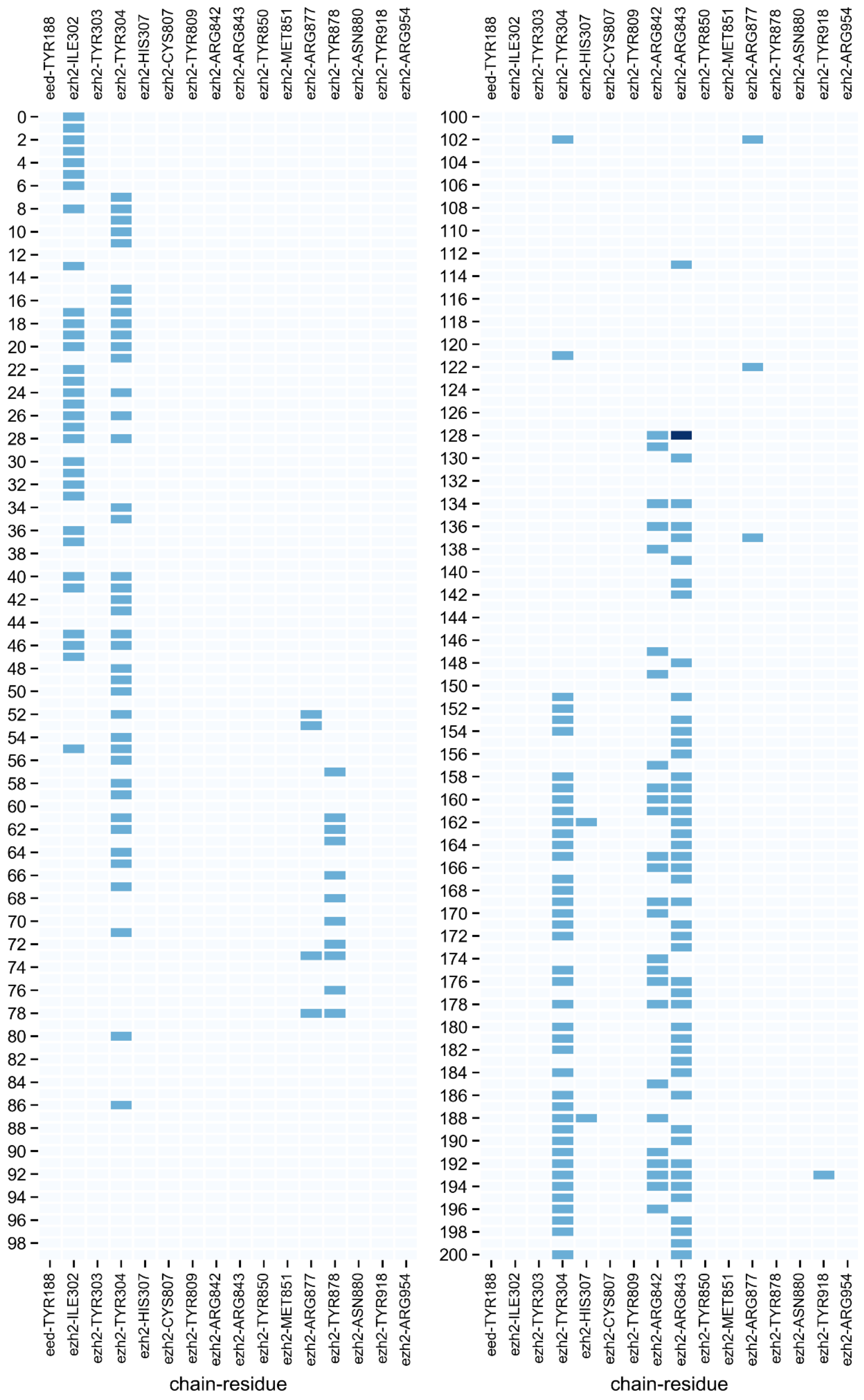

Supporting Figure S9. Hydrogen bonds established by CHEMBL3771372 with EZH2.

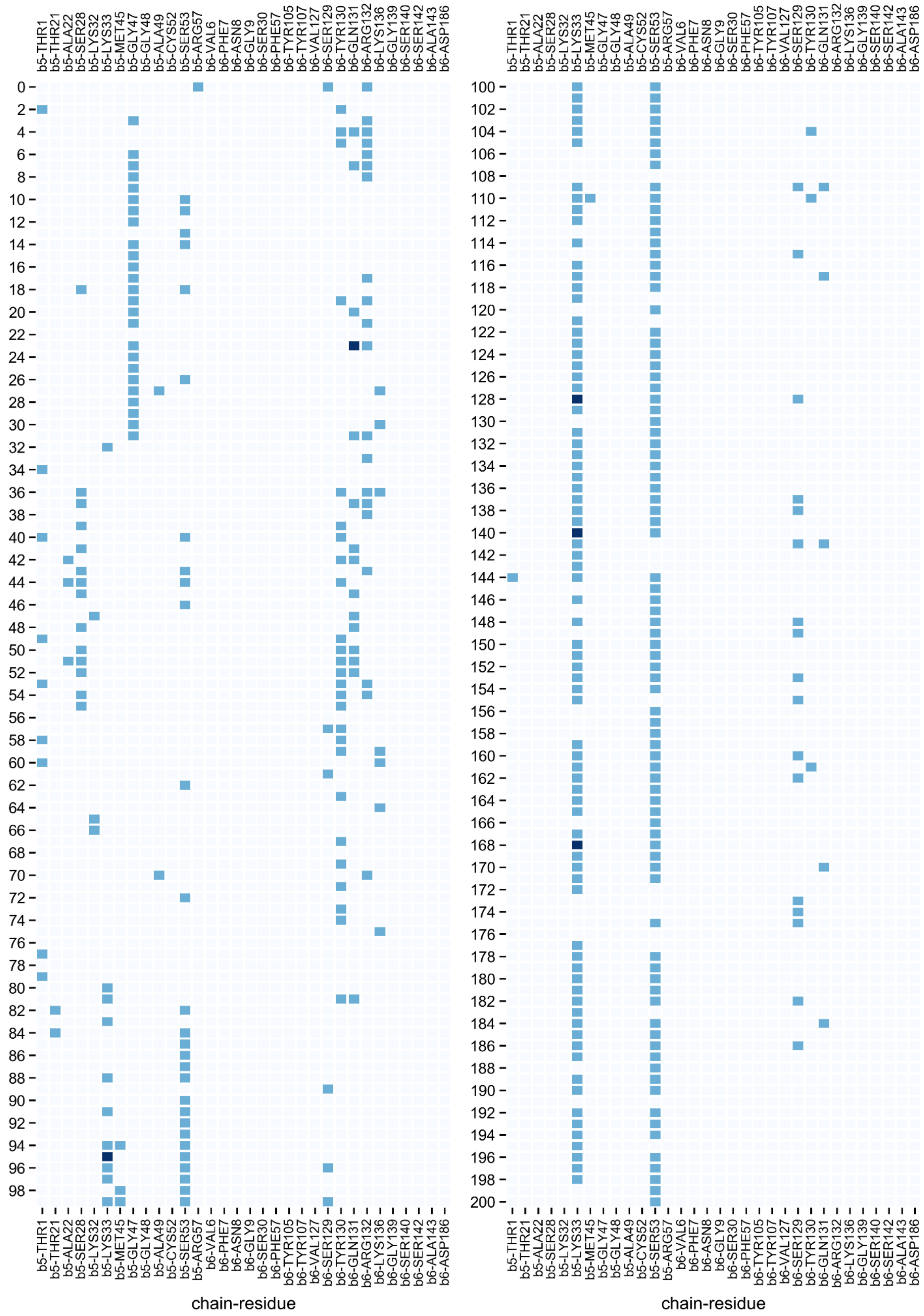

Supporting Figure S10. Hydrogen bonds established by CHEMBL3794075 with P20S.

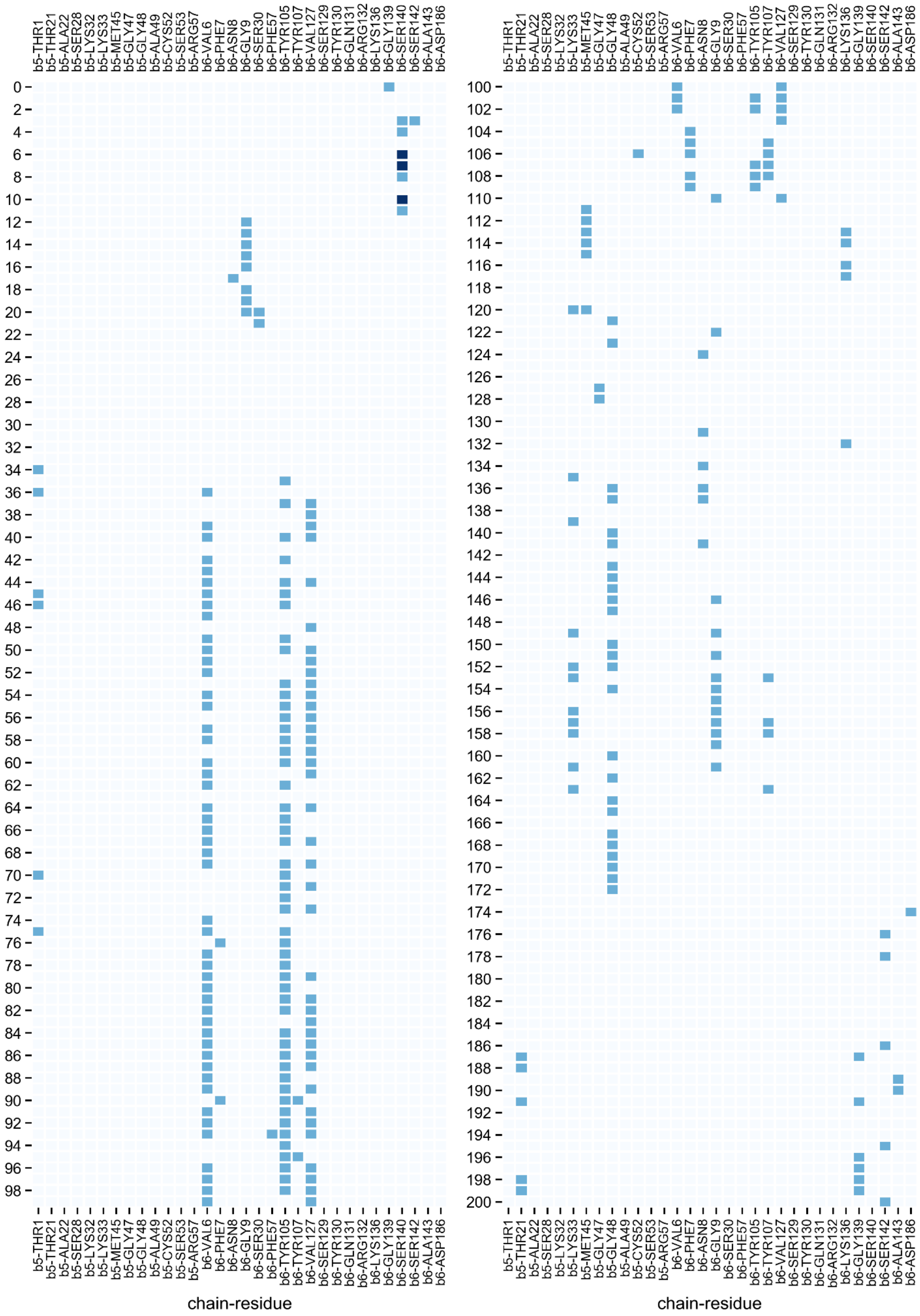

Supporting Figure S11. Hydrogen bonds established by CHEMBL3771372 with P20S.
